# Supplementary material for: Teratoma Assay for Testing Pluripotency and Malignancy of Stem Cells: Insufficient Reporting and Uptake of Animal-Free Methods—A Systematic Review
Source: Int J Mol Sci. 2023 Feb 15;24(4):3879. doi: 10.3390/ijms24043879 (PMC9967860; doi:10.3390/ijms24043879)
Supplement: Supplementary file 1 [file ijms-24-03879-s001.zip › ijms-2150070-supplementary.pdf]

**All supplementary figures and tables**

A

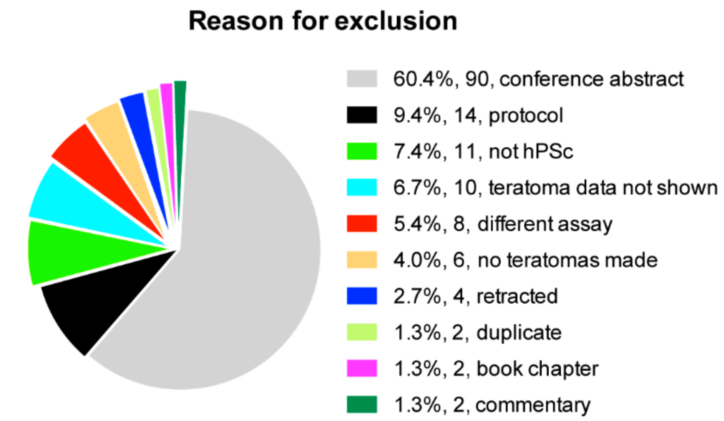


B

| Inclusion criteria not met | n |
|---|---|
| Cells other than hPSc | 5 |
| Nr. of injected cells not reported | 91 |
| Injection site not reported | 21 |
| Length of follow-up not reported | 55 |

**Figure S1.** Detailed information on exclusion and inclusion criteria. (**A**) Reasons for exclusion based on exclusion criteria. (**B**) Numbers of excluded publications based on non-met inclusion criteria.

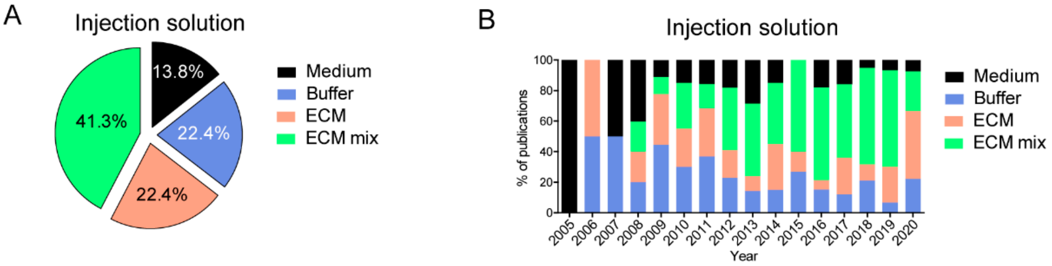


C

| Injection solution | Injection site: *Subcutaneous* Volume | % (n) | *Intramuscular* Volume | % (n) | *Organ* Volume | % (n) | *Other* Volume | % (n) |
|---|---|---|---|---|---|---|---|---|
| Medium | 10-400 µl | 7.9 (11) | 50-100 µl | 19.2 (5) | 20-200 µl | 21.4 (9) | 20-100 µl | 13.3 (2) |
| Buffer | 20-200 µl | 10.0 (14) | 30-300 µl | 19.2 (5) | 20-100 µl | 38.1 (16) | 30-100 µl | 40.0 (6) |
| ECM | 300-1000 µl | 13.6 (19) | 30-150 µl | 11.5 (3) | 10-40 µl | 19.0 (8) | 50-100 µl | 26.7 (4) |
| ECM mix | 20-200 µl | 68.6 (96) | 30-500 µl | 50.0 (13) | 10-200 µl | 21.4 (9) | 50-300 µl | 20.0 (3) |

**Figure S2.** Injection solution/medium, volume and injection site. (**A**) Pie diagram of solution types. (**B**)Percentages over years (2005-2020) in use of injection solutions. (**C**)Volumes used for injections depending on the injection site. Medium -culture medium; ECM - extracellular matrix such as Matrigel, collagen, alginate; buffer – PBS, DPBS, HBSS; ECM mix - mixture of ECM with either buffer or medium.

**Table S1.** Search strategies for inspected databases.

| | |
|---|---|
| **PubMed** | ("Teratoma"[Mesh:NoExp] OR "Teratoma"[tiab] OR "Teratomas"[tiab] OR "TA"[ti]) AND ("Stem Cells"[Majr:NoExp] OR "Stem Cell Transplantation"[Mesh] OR "Pluripotent Stem Cells"[Majr] OR "Stem Cell"[ti] OR "Stem Cells"[ti] OR "hESC"[ti] OR "hESCs"[ti] OR "hPSC"[ti] OR "hPCSs"[ti] OR "iPSC"[ti] OR "iPSCs"[ti] OR "iPS"[ti] OR "iPSs"[ti] OR "hiPSC"[ti] OR "hiPSCs"[ti]) AND ("malignancy"[tiab] OR "malignant"[tiab] OR "malignancies"[tiab] OR "tumorigenicity"[tiab] OR "tumorigenic"[tiab] **OR "tumourigenicity"[tiab] OR "tumourigenic"[tiab] OR** "pluripotency"[tiab] OR "pluripotent"[tiab] OR "Cell Differentiation"[Mesh:NoExp] OR "Cellular Reprogramming"[Mesh] OR "reprogramming"[tiab] OR "reprogram"[tiab] OR "reprogrammed"[tiab] OR "differentiation"[tiab] OR "differentiations"[tiab] OR "differentiated"[tiab] OR "differentiating"[tiab] OR "differentiate"[tiab] OR "undifferentiated"[tiab] OR "non-differentiated"[tiab]) NOT (("Swine"[Mesh] OR "Ruminants"[Mesh] OR "Rats"[Mesh] OR "Rabbits"[Mesh] OR "Cercopithecidae"[Mesh] OR "Canidae"[Mesh] OR "Mice"[Mesh] OR "Mouse Embryonic Stem Cells"[Mesh] OR "Book Reviews" [Publication Type] OR "Books"[Mesh] OR "Guideline" [Publication Type]) NOT "humans"[mesh]) NOT “Review” |
| **Embase** | ('teratoma'/exp OR 'teratoma':ti,ab OR 'teratomas':ti,ab) AND ('embryonic stem cell'/exp OR 'embryonic stem cell' OR 'embryonic stem cell*' OR 'human embryonic stem cells'/exp OR 'human embryonic stem cells' OR 'human embryonic stem cell*' OR 'pluripotent stem cell'/exp OR 'pluripotent stem cell' OR 'pluripotent stem cell*' OR 'totipotent stem cell'/exp OR 'totipotent stem cell' OR 'totipotent stem cell*' OR 'stem cell'/exp OR 'stem cell' OR 'stem cell*':ti,ab OR 'stem cell transplantation'/exp OR 'stem cell transplantation' OR 'hesc*':ti,ab OR 'ipsc*':ti,ab OR 'hipsc*' OR 'ips*':ti,ab) AND ('malignancy':ti,ab OR 'malignant':ti,ab OR 'maliganacies':ti,ab OR 'tumorigenicity':ti,ab OR 'tumorigenic':ti,ab OR 'tumourigenicity':ti,ab OR 'tumourigenic':ti,ab OR 'pluripotency':ti,ab OR 'pluripotent':ti,ab OR 'cell differentiation':ti,ab OR 'nuclear reprogramming':ti,ab OR 'reprogramming':ti,ab OR 'reprogram':ti,ab OR 'reprogrammed':ti,ab OR 'differentiation':ti,ab OR 'differentiations':ti,ab OR 'differentiated':ti,ab OR 'differentiating':ti,ab OR 'differentiate':ti,ab OR 'undifferentiated':ti,ab OR 'non-differentiated':ti,ab) NOT ('pig'/exp OR 'pig' OR 'ruminant'/exp OR 'ruminant' OR 'murine'/exp OR 'murine' OR 'cercopithecidae'/exp OR 'cercopithecidae' OR 'canidae'/exp OR 'canidae' OR 'mouse embryonic stem cell'/exp OR 'mouse embryonic stem cell' OR 'book editorial' OR 'letter'/exp OR 'letter' OR 'note'/exp OR 'note' OR 'review'/exp OR 'review' OR 'practice guideline'/exp OR 'practice guideline') AND [1998-2020]/py AND ([embase]/lim OR [medline]/lim OR [pubmed-not-medline]/lim) |
| **Web Of Science** | TS=("Teratoma" OR "Teratomas" OR "TA") AND TI=("stem cell" OR "Stem Cells" OR "hESC" OR "hESCs" OR "hPSC" OR "hPCSs" OR "iPSC" OR "iPSCs" OR "iPS" OR "iPSs" OR "hiPSC" OR "hiPSCs") AND TS=("malignancy" OR "malignant" OR "malignancies" OR "tumorigenicity" OR "tumorigenic" OR "tumourigenicity" OR "tumourigenic" OR "pluripotency" OR "pluripotent" OR "Cell Differentiation" OR "reprogramming" OR "reprogram" OR "reprogrammed" OR "differentiation" OR "differentiations" OR "differentiated" OR "differentiating" OR "differentiate" OR "undifferentiated" OR "non-differentiated") NOT TS=(("pig" OR "pigs" OR "porcine" OR "ruminant" OR "ruminants" OR "bovine" OR "murine" OR "murines" OR "rat" OR "rats" OR "guinea pig" OR "guinea pigs" OR leporidae OR "rabbit" OR "rabbits" OR "Cercopithecidae" OR "monkey" OR "monkeys" OR "macaca" OR "macacae" OR "macaque" OR "marsupial" OR "Canidae" OR "canine" OR "feline" OR "Mouse Embryonic Stem Cell" OR book OR editorial OR letter OR note OR "review" OR "practice Guideline") NOT "human") |

**Table S2.** List of mouse strains reported.

| Mouse strain | nr of publications |
|---|---|
| SCID | 142 |
| NOD SCID | 101 |
| SCID beige | 53 |
| NSG | 40 |
| CB17 SCID | 21 |
| *nude* | 19 |
| NOD SCID IL2Rγ−/− | 19 |
| NOD.Cg-Prkdc<scid>Il2rgtm1Wjl/SzJ | 11 |
| NOD.CB17-Prkdc-<scid>/J | 7 |
| NU-Foxn1nu | 7 |
| Balb/C nude | 6 |
| CB17/GbmsTac-scid-bgDF N7 | 6 |
| CB17.Cg-PrkdcscidLystbg-J/Crl | 5 |
| *not given* | 4 |
| CB17 SCID beige | 3 |

<table>
<tr><td>SCID Rag2-/-/γc-/-</td><td>3</td></tr>
<tr><td>BALB/cAJcl-Foxn1nu</td><td>3</td></tr>
<tr><td>BALB/cMlac-nu</td><td>2</td></tr>
<tr><td>immunocompromised</td><td>2</td></tr>
<tr><td>NOD.Cg-Prkdc<scid> Il2rg<tm1Sug> Jict/Tac</td><td>2</td></tr>
<tr><td>NOD/ShiJic-scidJcl</td><td>2</td></tr>
<tr><td>NIH-Lystbg-JFoxn1nuBtkxid</td><td>2</td></tr>
<tr><td>SHO-PrkdcscidHrhr</td><td>2</td></tr>
<tr><td>NMRI-Foxn1nu</td><td>2</td></tr>
<tr><td>BALB/c athymic nude</td><td>1</td></tr>
<tr><td>BALB/c Rag2-/-/γc-/-</td><td>1</td></tr>
<tr><td>BALB/cSlc-nu</td><td>1</td></tr>
<tr><td>CB17/Icr-Prkdcscid/CrlCrlj</td><td>1</td></tr>
<tr><td>NOD MrkBomTac-Prkdcscid</td><td>1</td></tr>
<tr><td>NOD SCID and nude</td><td>1</td></tr>
<tr><td>NOD.CB17Prkdcscid/JNarl</td><td>1</td></tr>
<tr><td>NOD/LtSz Prkd cscid</td><td>1</td></tr>
<tr><td>NOD/LtSz scid IL2R-/-</td><td>1</td></tr>
<tr><td>NOD/NCrCrl Prkd SCID</td><td>1</td></tr>
<tr><td>NSG & NOD-SCID</td><td>1</td></tr>
<tr><td>NSG SCID beige mice</td><td>1</td></tr>
<tr><td>Rag2-/-Il2rg-/-</td><td>1</td></tr>
<tr><td>Rag2−/−γc−/−</td><td>1</td></tr>
<tr><td>SCID CB17/Icr-Prkdcscid/CrlCrlj</td><td>1</td></tr>
<tr><td>SCID beige | NUDE</td><td>1</td></tr>
<tr><td>SCID beige | Balb/c scid</td><td>1</td></tr>
<tr><td>C.B-Igh-1bIcrTac-Prkdcscid/Ros</td><td>1</td></tr>
<tr><td>NOD.CB17-Prkdc<scid>/JSlac</td><td>1</td></tr>
<tr><td>NU(NCr)-Foxn1nu</td><td>1</td></tr>
<tr><td>nude | SCID</td><td>1</td></tr>
<tr><td>Rag2−/−γc−/−C2−/−</td><td>1</td></tr>
<tr><td>Rag2-/- | Pfp-/-/Rag2-/-</td><td>1</td></tr>
<tr><td>CD1-Foxn1nu</td><td>1</td></tr>
<tr><td>C.B-17/I cr-scid/scidJcl</td><td>1</td></tr>
<tr><td>Rag2−/−γc−/−C5−/−</td><td>1</td></tr>
<tr><td>C57Bl6 SCID</td><td>1</td></tr>
<tr><td>NOD MrkBomTac-Prkdcscid / NOD/LtSz Prkd cscid</td><td>1</td></tr>
<tr><td>Total publications</td><td>492</td></tr>
</table>

**List of references included in the systematic analysis**


1. Amit, M., et al., Clonally derived human embryonic stem cell lines maintain pluripotency and proliferative potential for prolonged periods of culture. Developmental biology, 2000. 227(2): p. 271-8.
2. Reubinoff, B.E., et al., Embryonic stem cell lines from human blastocysts: Somatic differentiation in vitro. Nat. Biotech0l., 2000. 18(4): p. 399-404.
3. Lebkowski, J.S., et al., Human embryonic stem cells: Culture, differentiation, and genetic modification for regenerative medicine applications. Cancer journal, 2001. 7: p. S83-S93.
4. Reubi0ff, B.E., et al., Effective cryopreservation of human embryonic stem cells by the open pulled straw vitrification method. Human reproduction (Oxford, England), 2001. 16(10): p. 2187-94.
5. Xu, C., et al., Feeder-free growth of undifferentiated human embryonic stem cells. Nature biotechnology, 2001. 19(10): p. 971-4.
6. Richards, M., et al., Human feeders support prolonged undifferentiated growth of human inner cell masses and embryonic stem cells. Vol. 20. 2002, United States. 933-6.
7. Amit, M., et al., Human feeder layers for human embryonic stem cells. Biology and reproduction, 2003. 68, 2150–2156
8. Gropp, M., et al., Stable genetic modification of human embryonic stem cells by lentiviral vectors. Molecular therapy : the journal of the American Society of Gene Therapy, 2003. 7(2): p. 281-7.
9. Hovatta, O., et al., A culture system using human foreskin fibroblasts as feeder cells allows production of human embryonic stem cells. Human reproduction (Oxford, England), 2003. 18(7): p. 1404-9.
10. Richards, M., et al., Comparative evaluation of various human feeders for prolonged undifferentiated growth of human embryonic stem cells. Vol. 21. 2003, United States. 546-56.
11. Tzukerman, M., et al., An experimental platform for studying growth and invasiveness of tumor cells within teratomas derived from human embryonic stem cells. PNAS, 2003. 100(23) 13507-13512.
12. Amit, M., et al., Feeder Layer- and Serum-Free Culture of Human Embryonic Stem Cells. Biology and reproduction, 2004. 70, 837–845.
13. Choo, A.B., et al., Expansion of pluripotent human embryonic stem cells on human feeders. Vol. 88. 2004, United States. 321-31.
14. Gerecht-Nir, S., et al., Vascular development in early human embryos and in teratomas derived from human embryonic stem cells. Biology of reproduction, 2004. 71(6): p. 2029-36.
15. Gertow, K., et al., Organized development from human embryonic stem cells after injection into immunodeficient mice. Stem cells and development, 2004. 13(4): p. 421-35.
16. Lavon, N., O. Yanuka, and N. Benvenisty, Differentiation and isolation of hepatic-like cells from human embryonic stem cells. Differentiation; research in biological diversity, 2004. 72(5): p. 230-8.
17. Liu, Y.P., et al., Maintenance of pluripotency in human embryonic stem cells stably over-expressing enhanced green fluorescent protein. Stem Cells Dev., 2004. 13(6): p. 636-645.
18. Rosler, E.S., et al., Long-term culture of human embryonic stem cells in feeder-free conditions. Developmental dynamics : an official publication of the American Association of Anatomists, 2004. 229(2): p. 259-74.
19. Stojkovic, M., et al., Derivation of human embryonic stem cells from day-8 blastocysts recovered after three-step in vitro culture. Stem cells (Dayton, Ohio), 2004. 22(5): p. 790-7.
20. Suss-Toby, E., et al., Derivation of a diploid human embryonic stem cell line from a mononuclear zygote. Human reproduction, 2004. 19(3) 670-675.
21. Fang, Z.F., et al., Human embryonic stem cell lines derived from the Chinese population. Cell research, 2005. 15(5): p. 394-400.
22. Fong, W.J., et al., Perfusion cultures of human embryonic stem cells. Bioprocess and biosystems engineering, 2005. 27(6): p. 381-387.
23. Inzunza, J., et al., Derivation of human embryonic stem cell lines in serum replacement medium using postnatal human fibroblasts as feeder cells. Stem cells (Dayton, Ohio), 2005. 23(4): p. 544-9.
24. Klimanskaya, I., et al., Human embryonic stem cells derived without feeder cells. Lancet (London, England), 2005. 365(9471): p. 1636-41.
25. Sjögren-Jansson, E., et al., Large-scale propagation of four undifferentiated human embryonic stem cell lines in a feeder-free culture system. Developmental dynamics : an official publication of the American Association of Anatomists, 2005. 233(4): p. 1304-14.
26. Stojkovic, P., et al., An autogeneic feeder cell system that efficiently supports growth of undifferentiated human embryonic stem cells. Stem Cells, 2005. 23(3): p. 306-314.
27. Wang, L., et al., Human embryonic stem cells maintained in the absence of mouse embryonic fibroblasts or conditioned media are capable of hematopoietic development. Blood, 2005. 105(12): p. 4598-603.

28. Wu, C.F., et al., Improved cryopreservation of human embryonic stem cells with trehalose. Reproductive biomedicine online, 2005. 11(6): p. 733-9.
29. Xiong, C., et al., Genetic engineering of human embryonic stem cells with lentiviral vectors. Stem cells and development, 2005. 14(4): p. 367-77.
30. Chen, S., et al., TFG-β2 allows pluripotent human embryonic stem cell proliferation on E6/E7 immortalized mouse embryonic fibroblasts. Journal of Biotechnology, 2006. 122 (341–361)
31. Choo, A., et al. Immortalized feeders for the scale-up of human embryonic stem cells in feeder and feeder-free conditions. J Biotechnology, 2006. 122(1):130-41
32. Cooke, M.J., M. Stojkovic, and S.A. Przyborski, Growth of teratomas derived from human pluripotent stem cells is influenced by the graft site. Stem cells and development, 2006. 15(2): p. 254-9.
33. Dean, S.K., et al., Differentiation of encapsulated embryonic stem cells after transplantation. Transplantation, 2006. 82(9): p. 1175-84.
34. Hong-mei, P. and C. Gui-an, Serum-free medium cultivation to improve efficacy in establishment of human embryonic stem cell lines. Vol. 21. 2006, England. 217-22.
35. Lavon, N., O. Yanuka, and N. Benvenisty, The effect of overexpression of Pdx1 and Foxa2 on the differentiation of human embryonic stem cells into pancreatic cells. Stem cells (Dayton, Ohio), 2006. 24(8): p. 1923-30.
36. Li, S.S.L., et al., Characterization and gene expression profiling of five new human embryonic stem cell lines derived in Taiwan. Stem cells and development, 2006. 15(4): p. 532-555.
37. Lu, J., et al., Defined culture conditions of human embryonic stem cells. Proceedings of the National Academy of Sciences of the United States of America, 2006. 103(15): p. 5688-93.
38. Mikkola, M., et al., Distinct differentiation characteristics of individual human embryonic stem cell lines. Vol. 6. 2006. 40.
39. Plaia, T.W., et al., Characterization of a new NIH-registered variant human embryonic stem cell line, BG01V: A tool for human embryonic stem cell research. Stem Cells, 2006. 24(3): p. 531-546.
40. Sidhu, K.S. and B.E. Tuch, Derivation of three clones from human embryonic stem cell lines by FACS sorting and their characterization. Vol. 15. 2006, United States. 61-9.
41. Suemori, H., et al., Efficient establishment of human embryonic stem cell lines and long-term maintenance with stable karyotype by enzymatic bulk passage. Biochemical and biophysical research communications, 2006. 345(3): p. 926-32.
42. Valbuena, D., et al., Derivation and characterization of three new Spanish human embryonic stem cell lines (VAL -3 -4 -5) on human feeder and in serum-free conditions. Reproductive biomedicine online, 2006. 13(6): p. 875-86.
43. Yao, S., et al., Long-term self-renewal and directed differentiation of human embryonic stem cells in chemically defined conditions. Proceedings of the National Academy of Sciences of the United States of America, 2006. 103(18): p. 6907-12.
44. Blum, B. and N. Benvenisty, Clonal analysis of human embryonic stem cell differentiation into teratomas. Stem cells (Dayton, Ohio), 2007. 25(8): p. 1924-30.
45. Brimble, S.N., et al., The cell surface glycosphingolipids SSEA-3 and SSEA-4 are not essential for human ESC pluripotency. Stem Cells, 2007. 25(1): p. 54-62.
46. Crook, J.M., et al., The generation of six clinical-grade human embryonic stem cell lines. Vol. 1. 2007, United States. 490-4.
47. Eiges, R., et al., Developmental study of fragile X syndrome using human embryonic stem cells derived from preimplantation genetically diagnosed embryos. Cell stem cell, 2007. 1(5): p. 568-77.
48. Gertow, K., et al., Trisomy 12 in HESC leads to 0 selective in vivo growth advantage in teratomas, but induces an increased abundance of renal development. Journal of cellular biochemistry, 2007. 100(6): p. 1518-25.
49. Gharwan, H., et al., Transduction of human embryonic stem cells by foamy virus vectors. Molecular therapy : the journal of the American Society of Gene Therapy, 2007. 15(10): p. 1827-33.
50. Kim, S., et al., A novel culture technique for human embryonic stem cells using porous membranes. Vol. 25. 2007, United States. 2601-9.
51. Lin, G., et al., A highly homozygous and parthenogenetic human embryonic stem cell line derived from a one-pronuclear oocyte following in vitro fertilization procedure. Cell research, 2007. 17(12): p. 999-1007.
52. Mai, Q., et al., Derivation of human embryonic stem cell lines from parthenogenetic blastocysts. Cell research, 2007. 17(12): p. 1008-19.
53. Masaki, H., et al., Heterogeneity of pluripotent marker gene expression in colonies generated in human iPS cell induction culture. Stem cell research, 2007. 1(2): p. 105-15.
54. Revazova, E.S., et al., Patient-specific stem cell lines derived from human parthenogenetic blastocysts. Cloning and stem cells, 2007. 9(3): p. 432-49.

55. Shih, C.C., et al., Human embryonic stem cells are prone to generate primitive, undifferentiated tumors in engrafted human fetal tissues in severe combined immunodeficient mice. Stem cells and development, 2007. 16(6): p. 893-902.
56. Ström, S., et al., Mechanical isolation of the inner cell mass is effective in derivation of new human embryonic stem cell lines. Human reproduction (Oxford, England), 2007. 22(12): p. 3051-8.
57. Takahashi, K., et al., Induction of Pluripotent Stem Cells from Adult Human Fibroblasts by Defined Factors. Cell, 2007. 131(5): p. 861-872.
58. Tan, S.M., et al., A UTF1-based selection system for stable homogeneously pluripotent human embryonic stem cell cultures. Nucleic acids research, 2007. 35(18): p. e118.
59. Wilber, A., et al., Efficient and stable transgene expression in human embryonic stem cells using transposon-mediated gene transfer. Vol. 25. 2007, United States. 2919-27.
60. Yu, J., et al., Induced pluripotent stem cell lines derived from human somatic cells. Science (New York, N.Y.), 2007. 318(5858): p. 1917-20.
61. Aasen, T., et al. Efficient and rapid generation of induced pluripotent stem cells from human keratinocytes. Nat Biotechnology, 2008. 26(11):1276-84
62. Chavez, SL., et al., Characterization of six new human embryonic stem cell lines (HSF7, -8, -9, -10, -12, and -13) derived under minimal-animal component conditions. Stem Cells Dev, 2008. 17(3): p. 535-46.
63. Choo, A.B., et al., Selection against undifferentiated human embryonic stem cells by a cytotoxic antibody recognizing podocalyxin-like protein-1. Stem cells (Dayton, Ohio), 2008. 26(6): p. 1454-63.
64. Feki, A., et al., Derivation of the first Swiss human embryonic stem cell line from a single blastomere of an arrested four-cell stage embryo. Swiss medical weekly, 2008. 138(37): p. 540-50.
65. Gjerstorff, M.F., et al., Distinct GAGE and MAGE-A expression during early human development indicate specific roles in lineage differentiation. Hum. Reprod., 2008. 23(10): p. 2194-2201.
66. Grinnemo, K.H., et al., Costimulation blockade induces tolerance to HESC transplanted to the testis and induces regulatory T-cells to HESC transplanted into the heart. Stem cells (Dayton, Ohio), 2008. 26(7): p. 1850-7.
67. Harb, N., et al., The Rho-Rock-Myosin Signaling Axis Determines Cell-Cell Integrity of Self-Renewing Pluripotent Stem Cells. Plos one, 2008. 3, e3001.
68. Kwok-Keung Chan, K., et al., Generation of high-level stable transgene expressing human embryonic stem cell lines using Chinese hamster elongation factor-1$\alpha$ promoter system. Stem Cells Dev., 2008. 17(4): p. 825-836.
69. Li, Z., et al., Comparison of reporter gene and iron particle labeling for tracking fate of human embryonic stem cells and differentiated endothelial cells in living subjects. Vol. 26. 2008. 864-73.
70. Park, I.H., et al., Reprogramming of human somatic cells to pluripotency with defined factors. Nature, 2008. 451(7175): p. 141-6.
71. Raya, A., et al. Generation of cardiomyocytes from new human embryonic stem cell lines derived from poor-quality blastocysts. Cold Spring Harb Symp Quant Biol, 2008. 73:127-35
72. Séguin, C.A., et al., Establishment of endoderm progenitors by SOX transcription factor expression in human embryonic stem cells. Cell stem cell, 2008. 3(2): p. 182-95.
73. Unger, C., et al., Lentiviral-mediated HoxB4 expression in human embryonic stem cells initiates early hematopoiesis in a dose-dependent manner but does not promote myeloid differentiation. Stem cells (Dayton, Ohio), 2008. 26(10): p. 2455-66.
74. Valbuena, D., et al., Efficient method for slow cryopreservation of human embryonic stem cells in xeno-free conditions. Reproductive biomedicine online, 2008. 17(1): p. 127-35.
75. Yang, C., et al., A key role for telomerase reverse transcriptase unit in modulating human embryonic stem cell proliferation, cell cycle dynamics, and in vitro differentiation. Stem Cells, 2008. 26(4): p. 850-863.
76. Yang, S., et al., Tumor progression of culture-adapted human embryonic stem cells during long-term culture. Genes, chromosomes & cancer, 2008. 47(8): p. 665-79.
77. Zhan, X., et al., Cells derived from human umbilical cord blood support the long-term growth of undifferentiated human embryonic stem cells. Cloning and stem cells, 2008. 10(4): p. 513-22.
78. Bigdeli, N., et al., Coculture of Human Embryonic Stem Cells and Human Articular Chondrocytes Results in Significantly Altered Phenotype and Improved Chondrogenic Differentiation. Stem cells, 2009. 27(8): p. 1812-1821.
79. Blum, B., et al., The anti-apoptotic gene survivin contributes to teratoma formation by human embryonic stem cells. Nature biotechnology, 2009. 27(3): p. 281-7.
80. De Sousa, P., et al. Clinically failed eggs as a source of normal human embryo stem cells. Stem cell Research, 2009.
81. Geens, M., et al., Human embryonic stem cell lines derived from single blastomeres of two 4-cell stage embryos. Human reproduction (Oxford, England), 2009. 24(11): p. 2709-17.

82. Harkness, L., et al., Isolation and differentiation of chondrocytic cells derived from human embryonic stem cells using dlk1/FA1 as a novel surface marker. Stem cell reviews and reports, 2009. 5(4): p. 353-68.
83. Hentze, H., et al., Teratoma formation by human embryonic stem cells: Evaluation of essential parameters for future safety studies. Stem cell research, 2009. 2(3): p. 198-210.
84. Hotta, A., et al., Isolation of human iPS cells using EOS lentiviral vectors to select for pluripotency. Nature methods, 2009. 6(5): p. 370-6.
85. Inamdar, M.S., et al., Derivation and Characterization of Two Sibling Human Embryonic Stem Cell Lines From Discarded Grade III Embryos. Stem cells and development, 2009. 18(3): p. 423-433.
86. Ji, J., et al., Pluripotent transcription factors possess distinct roles in normal versus transformed human stem cells. PloS one, 2009. 4(11): p. e8065.
87. King, F.W., et al., Subpopulations of human embryonic stem cells with distinct tissue-specific fates can be selected from pluripotent cultures. Stem cells and development, 2009. 18(10): p. 1441-50.
88. Kumar, N., et al., Derivation and Characterization of Two Genetically Unique Human Embryonic Stem Cell Lines on In-House-Derived Human Feeders. Stem cells and development, 2009. 18(3): p. 435-445.
89. Lee, A.S., et al., Effects of cell number on teratoma formation by human embryonic stem cells. Cell cycle (Georgetown, Tex.), 2009. 8(16): p. 2608-12.
90. Li, C., et al., Pluripotency can be rapidly and efficiently induced in human amniotic fluid-derived cells. Vol. 18. 2009, England. 4340-9.
91. Liu, J., et al., Stable transgene expression in human embryonic stem cells after simple chemical transfection. Molecular reproduction and development, 2009. 76(6): p. 580-6.
92. Nagata, S., et al., Efficient reprogramming of human and mouse primary extra-embryonic cells to pluripotent stem cells. Genes to cells : devoted to molecular & cellular mechanisms, 2009. 14(12): p. 1395-404.
93. Nakahara, M., et al., Human Embryonic Stem Cells with Maintenance under a Feeder-Free and Recombinant Cytokine-Free Condition. Cloning and stem cells, 2009. 11(1): p. 5-17.
94. Pomper, M.G., et al., Serial imaging of human embryonic stem-cell engraftment and teratoma formation in live mouse models. Cell research, 2009. 19(3): p. 370-9.
95. Priddle, H., et al., Bioluminescence imaging of human embryonic stem cells transplanted in vivo in murine and chick models. Cloning and stem cells, 2009. 11(2): p. 259-67.
96. Prokhorova, T. A., et al., Teratoma formation by human embryonic stem cells is site dependent and enhanced by the presence of Matrigel. Stem Cells and Development, 2009. 18(1).
97. Raya, A., et al. Disease-corrected haematopoietic progenitors from Fanconi anaemia induced pluripotent stem cells. Nature, 2009. 460(7251):53-9
98. Suhr, S.T., et al., Telomere dynamics in human cells reprogrammed to pluripotency. PLoS ONE, 2009. 4(12).
99. Toh, W.S., et al., Differentiation and enrichment of expandable chondrogenic cells from human embryonic stem cells in vitro. Journal of cellular and molecular medicine, 2009. 13(9): p. 3570-90.
100. Urbach, A. and N. Benvenisty, Studying early lethality of 45,XO (Turner's syndrome) embryos using human embryonic stem cells. PloS one, 2009. 4(1): p. e4175.
101. Utikal, J., et al., Sox2 is dispensable for the reprogramming of melanocytes and melanoma cells into induced pluripotent stem cells. Journal of cell science, 2009. 122: p. 3502-10.
102. Ware, C.B., et al., Histone deacetylase inhibition elicits an evolutionarily conserved self-renewal program in embryonic stem cells. Cell stem cell, 2009. 4(4): p. 359-69.
103. Werbowetski-Ogilvie, T.E., et al., Characterization of human embryonic stem cells with features of neoplastic progression. Nature biotechnology, 2009. 27(1): p. 91-7.
104. Yu, J., et al. Human Induced Pluripotent Stem Cells Free of Vector and Transgene Sequences. Science, 2009. 324(5928):797-801
105. Zhou, W. and C.R. Freed, Ade0viral gene delivery can reprogram human fibroblasts to induced pluripotent stem cells. Stem cells (Dayton, Ohio), 2009. 27(11): p. 2667-74.
106. Aguilar-Gallardo, C., et al., Derivation, characterization, differentiation, and registration of seven human embryonic stem cell lines (VAL-3, -4, -5, -6M, -7, -8, and -9) on human feeder. In Vitro Cell. Dev. Biol. Anim., 2010. 46(3): p. 317-326.
107. Armstrong, L., et al., Human induced pluripotent stem cell lines show stress defense mechanisms and mitochondrial regulation similar to those of human embryonic stem cells. Stem cells (Dayton, Ohio), 2010. 28(4): p. 661-73.
108. Bradley, C.K., et al., Derivation of three new human embryonic stem cell lines. In vitro cellular & developmental biology. Animal, 2010. 46(3): p. 294-9.
109. Cai, J.L., et al., Generation of Human Induced Pluripotent Stem Cells from Umbilical Cord Matrix and Amniotic Membrane Mesenchymal Cells. Journal of biological chemistry, 2010. 285(15): p. 11227-11234.
110. Cao, F., et al., Comparison of gene-transfer efficiency in human embryonic stem cells. Vol. 12. 2010. 15-24.

111. Carette, J.E., et al., Generation of hiPSc from cultured human malignant cells. Blood, 2010. 115(20): p. 4039-42.
112. Chayosumrit, M., B. Tuch, and K. Sidhu, Alginate microcapsule for propagation and directed differentiation of hESCs to definitive endoderm. Vol. 31. 2010, Netherlands. 505-14.
113. Chin, A.C.P., et al., Defined and Serum-Free Media Support Undifferentiated Human Embryonic Stem Cell Growth. Stem cells and development, 2010. 19(6): p. 753-761.
114. Du, J., et al., The combined use of viral transcriptional and post-transcriptional regulatory elements to improve baculovirus-mediated transient gene expression in human embryonic stem cells. Journal of bioscience and bioengineering, 2010. 109(1): p. 1-8.
115. Fu, X., et al., Autologous feeder cells from embryoid body outgrowth support the long-term growth of human embryonic stem cells more effectively than those from direct differentiation. Tissue engineering. Part C, Methods, 2010. 16(4): p. 719-33.
116. Fujioka, T., et al., Establishment of induced pluripotent stem cells from human neonatal tissues. Human cell, 2010. 23(3): p. 113-8.
117. Galende, E., et al., Amniotic fluid cells are more efficiently reprogrammed to pluripotency than adult cells. Cell. Reprogramming, 2010. 12(2): p. 117-125.
118. Gori, J.L., et al., In vivo selection of human embryonic stem cell-derived cells expressing methotrexate-resistant dihydrofolate reductase. Gene therapy, 2010. 17(2): p. 238-49.
119. Gutierrez-Aranda, I., et al., Human induced pluripotent stem cells develop teratoma more efficiently and faster than human embryonic stem cells regardless the site of injection. Vol. 28. 2010. 1568-70.
120. Hansel, M.C., et al., Generation of induced pluripotent stem cells from the hepatocytes of normal patients and patients with metabolic liver diseases. Hepatology, 2010. 52: p. 413A-414A.
121. Harkness, L., et al., Derivation and characterisation of hESC lines from supernumerary embryos, experience from Odense, Denmark. In vitro cellular & developmental biology. Animal, 2010. 46(3): p. 259-68.
122. Hayashi, Y., et al., Reduction of N-glycolylneuraminic acid in human induced pluripotent stem cells generated or cultured under feeder- and serum-free defined conditions. PloS one, 2010. 5(11): p. e14099.
123. Holm, F., et al., An effective serum- and xeno-free chemically defined freezing procedure for human embryonic and induced pluripotent stem cells. Vol. 25. 2010. 1271-9.
124. Hwang, S.T., et al., The expansion of human ES and iPS cells on porous membranes and proliferating human adipose-derived feeder cells. Biomaterials, 2010. 31(31): p. 8012-21.
125. Jones, M.B., et al., Proliferation and pluripotency of human embryonic stem cells maintained on type I collagen. Stem cells and development, 2010. 19(12): p. 1923-35.
126. Kazuki, Y., et al., Complete Genetic Correction of iPS Cells From Duchenne Muscular Dystrophy. Molecular therapy, 2010. 18(2): p. 386-393.
127. Lai, D., et al., Optimization of culture conditions to support undifferentiated growth of human embryonic stem cells. Cellular reprogramming, 2010. 12(3): p. 305-14.
128. Lai, W.H., et al., ROCK inhibition facilitates the generation of human-induced pluripotent stem cells in a defined, feeder-, and serum-free system. Cellular reprogramming, 2010. 12(6): p. 641-53.
129. Lee, E.J., et al., novel Embryoid Body-Based Method to Derive Mesenchymal Stem Cells from Human Embryonic Stem Cells. Tissue engineering part a, 2010. 16(2): p. 705-715.
130. Li, C., et al., Efficient derivation of Chinese human embryonic stem cell lines from frozen embryos. In vitro cellular & developmental biology. Animal, 2010. 46(3): p. 186-91.
131. Lin, S.A., et al., Subfractionation of differentiating human embryonic stem cell populations allows the isolation of a mesodermal population enriched for intermediate mesoderm and putative renal progenitors. Stem cells and development, 2010. 19(10): p. 1637-48.
132. Liu, H., et al., Generation of endoderm-derived human induced pluripotent stem cells from primary hepatocytes. Hepatology (Baltimore, Md.), 2010. 51(5): p. 1810-9.
133. Lu, H., et al., Efficient differentiation of newly derived human embryonic stem cells from discarded blastocysts into hepatocyte-like cells. Journal of digestive diseases, 2010. 11(6): p. 376-82.
134. Narwani, K., et al., Human embryonic stem cells from aneuploid blastocysts identified by pre-implantation genetic screening. In vitro cellular & developmental biology. Animal, 2010. 46(3): p. 309-16.
135. Ng, V.Y., et al., Characterization of Epithelial Cell Adhesion Molecule as a Surface Marker on Undifferentiated Human Embryonic Stem Cells. Stem cells, 2010. 28(1): p. 29-35.
136. Pryzhkova, M.V., A. Peters, and E.T. Zambidis, Erythropoietic differentiation of a human embryonic stem cell line harbouring the sickle cell anaemia mutation. Reproductive biomedicine online, 2010. 21(2): p. 196-205.
137. Rodin, S., et al., Long-term self-renewal of human pluripotent stem cells on human recombinant laminin-511. Nature biotechnology, 2010. 28(6): p. 611-5.

138. Rodríguez-Pizà, I., et al., Reprogramming of human fibroblasts to induced pluripotent stem cells under xeno-free conditions. Stem cells (Dayton, Ohio), 2010. 28(1): p. 36-44.
139. Ross, P.J., et al., Human-Induced Pluripotent Stem Cells Produced Under Xeno-Free Conditions. Stem cells and development, 2010. 19(8): p. 1221-1229.
140. Ruiz, S., et al., High-efficient generation of induced pluripotent stem cells from human astrocytes. PloS one, 2010. 5(12): p. e15526.
141. Si-Tayeb, K., et al., Generation of human induced pluripotent stem cells by simple transient transfection of plasmid DNA encoding reprogramming factors. BMC developmental biology, 2010. 10: p. 81.
142. Sidhu, K.S., et al., Derivation of a new human embryonic stem cell line, Endeavour-2, and its characterization. In vitro cellular & developmental biology-animal, 2010. 46(3): p. 269-275.
143. Takenaka, C., et al., Effective generation of iPS cells from CD34+ cord blood cells by inhibition of p53. Experimental hematology, 2010. 38(2): p. 154-62.
144. Terstegge, S., et al., Laser-Assisted photoablation of human pluripotent stem cells from differentiating cultures. Stem Cell Rev. Rep., 2010. 6(2): p. 260-269.
145. Venu, P., S. Chakraborty, and M.S. Inamdar, Analysis of long-term culture properties and pluripotent character of two sibling human embryonic stem cell lines derived from discarded embryos. In vitro cellular & developmental biology. Animal, 2010. 46(3): p. 200-5.
146. Wilson, K.D., et al., Effects of ionizing radiation on self-renewal and pluripotency of human embryonic stem cells. Cancer research, 2010. 70(13): p. 5539-48.
147. Wu, R., et al., Derivation, characterization and differentiation of a new human embryonic stem cell line from a Chinese hatched blastocyst assisted by a non-contact laser system. Human cell, 2010. 23(3): p. 89-102.
148. Xi, J., et al., Human fetal liver stromal cells that overexpress bFGF support growth and maintenance of human embryonic stem cells. PloS one, 2010. 5(12): p. e14457.
149. Yan, X., et al., iPS cells reprogrammed from human mesenchymal-like Stem/Progenitor cells of dental Tissue Origin. Stem Cells Dev., 2010. 19(4): p. 469-480.
150. Ye, L., et al., Generation of induced pluripotent stem cells using site-specific integration with phage integrase. Proceedings of the National Academy of Sciences of the United States of America, 2010. 107(45): p. 19467-72.
151. Alva, J.A., et al., Phosphatase and tensin homolog regulates the pluripotent state and lineage fate choice in human embryonic stem cells. Stem cells (Dayton, Ohio), 2011. 29(12): p. 1952-62.
152. Anchan, R.M., et al., Amniocytes can serve a dual function as a source of iPS cells and feeder layers. Human molecular genetics, 2011. 20(5): p. 962-74.
153. Anokye-Danso, F., et al., Highly efficient miRNA-mediated reprogramming of mouse and human somatic cells to pluripotency. Cell stem cell, 2011. 8(4): p. 376-88.
154. Ardehali, R., et al., Overexpression of BCL2 enhances survival of human embryonic stem cells during stress and obviates the requirement for serum factors. Proceedings of the National Academy of Sciences of the United States of America, 2011. 108(8): p. 3282-7.
155. Arpornmaeklong, P., M.J. Pressler, and P.H. Krebsbach, Phenotypic and differentiation stability of human embryonic stem cell-derived osteoblasts. Cells, tissues, organs, 2011. 194(2): p. 326-30.
156. Beltrão-Braga, P.C., et al., Feeder-free derivation of induced pluripotent stem cells from human immature dental pulp stem cells. Cell transplantation, 2011. 20(11): p. 1707-19.
157. Bradley, C.K., et al., Derivation of Huntington's Disease-Affected Human Embryonic Stem Cell Lines. Stem cells and development, 2011. 20(3): p. 495-502.
158. Chang, T., et al., Brief report: phenotypic rescue of induced pluripotent stem cell-derived motoneurons of a spinal muscular atrophy patient. Stem cells (Dayton, Ohio), 2011. 29(12): p. 2090-3.
159. Deuse, T., et al., Immunobiology of naive and genetically modified HLA-class-I-knockdown human embryonic stem cells. Journal of cell science, 2011. 124(17): p. 3029-3037.
160. Fu, X., et al., Establishment of Clinically Compliant Human Embryonic Stem Cells in an Autologous Feeder-Free System. Tissue engineering part c-methods, 2011. 17(9): p. 927-937.
161. Germanguz, I., et al., Molecular characterization and functional properties of cardiomyocytes derived from human inducible pluripotent stem cells. Journal of cellular and molecular medicine, 2011. 15(1): p. 38-51.
162. Gertow, K., et al., Early Events in Xenograft Development from the Human Embryonic Stem Cell Line HS181-Resemblance with an Initial Multiple Epiblast Formation. Plos one, 2011. 6(11).
163. Kim, H.O., et al., Selective Depletion of SSEA-3-and TRA-1-60-Positive Undifferentiated Human Embryonic Stem Cells by Magnetic Activated Cell Sorter (MACS). Tissue engineering and regenerative medicine, 2011. 8(2): p. 253-261.

164. King, F.W., et al., High-throughput tracking of pluripotent human embryonic stem cells with dual fluorescence resonance energy transfer molecular beacons. Stem cells and development, 2011. 20(3): p. 475-84.
165. Kol'tsova, A.M., et al., Comparative Characteristics of New Human Embryonic Stem Cell Lines SC5, SC6, SC7, and SC3a. Russian journal of developmental biology, 2011. 42(4): p. 212-225.
166. Krtolica, A., et al., GRO$\alpha$ regulates human embryonic stem cell self-renewal or adoption of a neuronal fate. Differentiation; research in biological diversity, 2011. 81(4): p. 222-32.
167. Kumazaki, T., et al., Establishment of human induced pluripotent stem cell lines from normal fibroblast TIG-1. Human cell, 2011. 24(2): p. 96-103.
168. Mandegar, M.A., et al., Functional human artificial chromosomes are generated and stably maintained in human embryonic stem cells. Human molecular genetics, 2011. 20(15): p. 2905-13.
169. Matsumura, K., et al., Effective vitrification of human induced pluripotent stem cells using carboxylated ε-poly-l-lysine. Cryobiology, 2011. 63(2): p. 76-83.
170. Mehta, A., et al., Pharmacological response of human cardiomyocytes derived from virus-free induced pluripotent stem cells. Cardiovascular research, 2011. 91(4): p. 577-86.
171. Nishigaki, T., et al., Highly efficient cryopreservation of human induced pluripotent stem cells using a dimethyl sulfoxide-free solution. The International journal of developmental biology, 2011. 55(3): p. 305-11.
172. Rajesh, D., et al., Human lymphoblastoid B-cell lines reprogrammed to EBV-free induced pluripotent stem cells. Blood, 2011. 118(7): p. 1797-800.
173. Ritner, C., et al., An Engineered Cardiac Reporter Cell Line Identifies Human Embryonic Stem Cell-Derived Myocardial Precursors. Plos one, 2011. 6(1).
174. Su, W., et al., Bioluminescence reporter gene imaging characterize human embryonic stem cell-derived teratoma formation. Journal of cellular biochemistry, 2011. 112(3): p. 840-8.
175. Tang, C., et al., An antibody against SSEA-5 glycan on human pluripotent stem cells enables removal of teratoma-forming cells. Nature biotechnology, 2011. 29(9): p. 829-34.
176. Tsutsui, H., et al., An optimized small molecule inhibitor cocktail supports long-term maintenance of human embryonic stem cells. Nature communications, 2011. 2: p. 167.
177. Wada, N., et al., Induced pluripotent stem cell lines derived from human gingival fibroblasts and periodontal ligament fibroblasts. Journal of periodontal research, 2011. 46(4): p. 438-47.
178. Wu, Z., et al., Derivation and characterization of human embryonic stem cell lines from the Chinese population. J. Genet. Genomics, 2011. 38(1): p. 13-20.
179. Xie, X., et al., Effects of long-term culture on human embryonic stem cell aging. Stem cells and development, 2011. 20(1): p. 127-38.
180. Yap, L.Y.W., et al., Defining a Threshold Surface Density of Vitronectin for the Stable Expansion of Human Embryonic Stem Cells. Tissue engineering part c-methods, 2011. 17(2): p. 193-207.
181. Zhang, K., et al., Utilization of human amniotic mesenchymal cells as feeder layers to sustain propagation of human embryonic stem cells in the undifferentiated state. Cellular reprogramming, 2011. 13(4): p. 281-8.
182. Bai, H., et al., Bcl-xL enhances single-cell survival and expansion of human embryonic stem cells without affecting self-renewal. Stem cell research, 2012. 8(1): p. 26-37.
183. Camnasio, S., et al., The first reported generation of several induced pluripotent stem cell lines from homozygous and heterozygous Huntington's disease patients demonstrates mutation related enhanced lysosomal activity. Neurobiology of disease, 2012. 46(1): p. 41-51.
184. Chen, K.G., et al., non-colony type mo0layer culture of human embryonic stem cells. Vol. 9. 2012. 237-48.
185. Chen, X., et al., Triploid and diploid embryonic stem cell lines derived from tripronuclear human zygotes. Journal of assisted reproduction and genetics, 2012. 29(8): p. 713-21.
186. Chung, J., et al., Thera0stic effect of serial manganese-enhanced magnetic resonance imaging of human embryonic stem cell derived teratoma. Magnetic resonance in medicine, 2012. 68(2): p. 595-599.
187. Deng, F., et al., Generation of induced pluripotent stem cells from human Tenon's capsule fibroblasts. Molecular vision, 2012. 18: p. 2871-81.
188. Ding, D.C., et al., Human Umbilical Cord Mesenchymal Stem Cells Support nontumorigenic Expansion of Human Embryonic Stem Cells. Cell transplantation, 2012. 21(7): p. 1515-1527.
189. Egashira, T., et al., Disease characterization using LQTS-specific induced pluripotent stem cells. 2012: England. p. 419-29.
190. Griscelli, F., et al., Malignant germ cell-like tumors, expressing Ki-1 antigen (CD30), are revealed during in vivo differentiation of partially reprogrammed human-induced pluripotent stem cells. The American journal of pathology, 2012. 180(5): p. 2084-96.
191. Gropp, M., et al., Standardization of the teratoma assay for analysis of pluripotency of human ES cells and biosafety of their differentiated progeny. PloS one, 2012. 7(9): p. e45532.

192. Heng, B.C., et al., Translating human embryonic stem cells from 2-dimensional to 3-dimensional cultures in a defined medium on laminin- and vitronectin-coated surfaces. Stem cells and development, 2012. 21(10): p. 1701-15.
193. Hu, J., et al., Immortalized mouse fetal liver stromal cells support growth and maintenance of human embryonic stem cells. Oncology reports, 2012. 28(4): p. 1385-91.
194. Jiang, Y., et al., Derivation and Functional Analysis of Patient-Specific Induced Pluripotent Stem Cells as an In Vitro Model of Chronic Granulomatous Disease. Stem cells, 2012. 30(4): p. 599-611.
195. Keil, M., et al., Transcriptional expression profile of cultured human embryonic stem cells in vitro and in vivo. In vitro cellular & developmental biology. Animal, 2012. 48(3): p. 165-74.
196. Levi, B., et al., In vivo directed differentiation of pluripotent stem cells for skeletal regeneration. Proceedings of the National Academy of Sciences of the United States of America, 2012. 109(50): p. 20379-84.
197. Liu, T., et al., Low microRNA-199a expression in human amniotic epithelial cell feeder layers maintains human-induced pluripotent stem cell pluripotency via increased leukemia inhibitory factor expression. Acta biochimica et biophysica Sinica, 2012. 44(3): p. 197-206.
198. Liu, T., et al., Human amniotic epithelial cell feeder layers maintain human iPS cell pluripotency via inhibited endogenous microRNA-145 and increased Sox2 expression. Experimental cell research, 2012. 318(4): p. 424-34.
199. Liu, T., et al., High efficiency of reprogramming $CD34^{+}$ cells derived from human amniotic fluid into induced pluripotent stem cells with Oct4. Stem cells and development, 2012. 21(12): p. 2322-32.
200. Lu, H.F., et al., A 3D microfibrous scaffold for long-term human pluripotent stem cell self-renewal under chemically defined conditions. Biomaterials, 2012. 33(8): p. 2419-30.
201. Mou, X., et al., Generation of disease-specific induced pluripotent stem cells from patients with different karyotypes of Down syndrome. Stem cell research & therapy, 2012. 3(2): p. 14.
202. Nagata, S., et al., Self-Renewal and Pluripotency Acquired through Somatic Reprogramming to Human Cancer Stem Cells. Plos one, 2012. 7(11).
203. Nayler, S., et al., Induced pluripotent stem cells from ataxia-telangiectasia recapitulate the cellular phenotype. Stem Cells Transl. Med., 2012. 1(7): p. 523-535.
204. Nomura, Y., et al., Human periodontal ligament Wbroblasts are the optimal cell source for induced pluripotent stem cells. Histochem. Cell Biol., 2012. 137(6): p. 719-732.
205. Ohnishi, H., et al., A comparative study of induced pluripotent stem cells generated from frozen, stocked bone marrow- and adipose tissue-derived mesenchymal stem cells. Vol. 6. 2012, England. 261-71.
206. Ovchinnikov, D.A., et al., Generation of a human embryonic stem cell line stably expressing high levels of the fluorescent protein mCherry. World journal of stem cells, 2012. 4(7): p. 71-9.
207. Panicker, L.M., et al., Induced pluripotent stem cell model recapitulates pathologic hallmarks of Gaucher disease. Proceedings of the National Academy of Sciences of the United States of America, 2012. 109(44): p. 18054-9.
208. Qu, X., et al., Induced pluripotent stem cells generated from human adipose-derived stem cells using a non-viral polycistronic plasmid in feeder-free conditions. PloS one, 2012. 7(10): p. e48161.
209. Rong, Z.L., et al., A Scalable Approach to Prevent Teratoma Formation of Human Embryonic Stem Cells. Journal of biological chemistry, 2012. 287(39): p. 32338-32345.
210. Sánchez, L., et al., Maintenance of human embryonic stem cells in media conditioned by human mesenchymal stem cells obviates the requirement of exogenous basic fibroblast growth factor supplementation. Tissue engineering. Part C, Methods, 2012. 18(5): p. 387-96.
211. Sánchez-Danés, A., et al., Efficient generation of A9 midbrain dopaminergic neurons by lentiviral delivery of LMX1A in human embryonic stem cells and induced pluripotent stem cells. Human gene therapy, 2012. 23(1): p. 56-69.
212. Shah, S.N., et al., HMGA1 Reprograms Somatic Cells into Pluripotent Stem Cells by Inducing Stem Cell Transcriptional Networks. PLoS ONE, 2012. 7(11).
213. Wang, X.M., et al., The gene expression profiles of induced pluripotent stem cells from individuals with childhood cerebral adre0leukodystrophy are consistent with proposed mechanisms of pathogenesis. Stem cell research & therapy, 2012. 3(5): p. 39.
214. Zhang, Y.S., et al., Derivation, culture and retinal pigment epithelial differentiation of human embryonic stem cells using human fibroblast feeder cells. Journal of assisted reproduction and genetics, 2012. 29(8): p. 735-44.
215. Zou, X.Y., et al., Establishment of transgene-free induced pluripotent stem cells reprogrammed from human stem cells of apical papilla for neural differentiation. Stem cell research & therapy, 2012. 3(5): p. 43.

216. Zucchelli, M., et al., In vivo differentiated human embryonic stem cells can acquire chromosomal aberrations more frequently than in vitro during the same period. Stem cells and development, 2012. 21(18): p. 3363-71.
217. Abad, M., et al., Reprogramming in vivo produces teratomas and iPS cells with totipotency features. Nature, 2013. 502, 340-345
218. Abujarour, R., et al., Optimized surface markers for the prospective isolation of high-quality hiPSCs using flow cytometry selection. Scientific reports, 2013. 3: p. 1179.
219. Amabile, G., et al., In vivo generation of transplantable human hematopoietic cells from induced pluripotent stem cells. Blood, 2013. 121(8): p. 1255-64.
220. Awe, J.P., et al., Generation and characterization of transgene-free human induced pluripotent stem cells and conversion to putative clinical-grade status. Stem cell research & therapy, 2013. 4(4): p. 87.
221. Ben-David, U., et al., Selective elimination of human pluripotent stem cells by an oleate synthesis inhibitor discovered in a high-throughput screen. Cell stem cell, 2013. 12(2): p. 167-79.
222. Ben-David, U., N. Nudel, and N. Benvenisty, Immunologic and chemical targeting of the tight-junction protein Claudin-6 eliminates tumorigenic human pluripotent stem cells. Nature communications, 2013. 4: p. 1992.
223. Chen, A., et al., Extracellular matrix free microcarrier cultures of human pluripotent stem cells induced by inhibition of rock-myosin ii signaling. Cytotherapy, 2013. 15(4): p. S5-S6.
224. Chen, I.P., et al., Induced Pluripotent Stem Cell Reprogramming by Integration-Free Sendai Virus Vectors from Peripheral Blood of Patients with Craniometaphyseal Dysplasia. Cellular reprogramming, 2013. 15(6): p. 503-513.
225. Gordeeva, O.F. and T.M. Niko0va, Development of experimental tumors formed by mouse and human embryonic stem and teratocarcinoma cells after subcutaneous and intraperitoneal transplantations into immunodeficient and immunocompetent mice. Vol. 22. 2013, United States. 1901-14.
226. Hämäläinen, R.H., et al., Tissue- and cell-type-specific manifestations of heteroplasmic mtDNA 3243A>G mutation in human induced pluripotent stem cell-derived disease model. Proceedings of the National Academy of Sciences of the United States of America, 2013. 110(38): p. E3622-30.
227. Heng, B.C., et al., MRNA transfection-based, feeder-free, induced pluripotent stem cells derived from adipose tissue of a 50-year-old patient. Metab. Eng., 2013. 18: p. 9-24.
228. Jiao, J., et al., Modeling Dravet syndrome using induced pluripotent stem cells (iPSCs) and directly converted neurons. Hum. Mol. Genet., 2013. 22(21): p. 4241-4252.
229. Khan, M., et al., Delivery of reprogramming factors into fibroblasts for generation of non-genetic induced pluripotent stem cells using a cationic bolaamphiphile as a non-viral vector. Biomaterials, 2013. 34(21): p. 5336-43.
230. Kunova, M., et al., Adaptation to Robust Mo0layer Expansion Produces Human Pluripotent Stem Cells With Improved Viability. Stem cells translational medicine, 2013. 2(4): p. 246-254.
231. Lee, M.O., et al., Inhibition of pluripotent stem cell-derived teratoma formation by small molecules. Proceedings of the National Academy of Sciences of the United States of America, 2013. 110(35): p. E3281-90.
232. Liang, Y., et al., The propensity for tumorigenesis in human induced pluripotent stem cells is related with genomic instability. Chinese journal of cancer, 2013. 32(4): p. 205-12.
233. Lu, H.E., et al., Modeling neurogenesis impairment in Down syndrome with induced pluripotent stem cells from Trisomy 21 amniotic fluid cells. Vol. 319. 2013, United States. 498-505.
234. Merling, R.K., et al., Transgene-free iPSCs generated from small volume peripheral blood nonmobilized CD34+ cells. Blood, 2013. 121(14)
235. Phang, R.Z., et al., Zinc finger nuclease-expressing baculoviral vectors mediate targeted genome integration of reprogramming factor genes to facilitate the generation of human induced pluripotent stem cells. Stem Cells Transl. Med., 2013. 2(12): p. 935-945.
236. Polanco, J.C., et al., Identification of unsafe human induced pluripotent stem cell lines using a robust surrogate assay for pluripotency. Stem cells (Dayton, Ohio), 2013. 31(8): p. 1498-510.
237. Roy, S., et al., Rare somatic cells from human breast tissue exhibit extensive lineage plasticity. Proc. Natl. Acad. Sci. U. S. A., 2013. 110(12): p. 4598-4603.
238. Sequiera, G.L., et al., Ontogenic development of cardiomyocytes derived from transgene-free human induced pluripotent stem cells and its homology with human heart. Life sciences, 2013. 92(1): p. 63-71.
239. Soyombo, A.A., et al., Analysis of induced pluripotent stem cells from a BRCA1 mutant family. Stem cell reports, 2013. 1(4): p. 336-49.
240. Stachelscheid, H., et al., Teratoma formation of human embryonic stem cells in three-dimensional perfusion culture bioreactors. J. Tissue Eng. Regenerative Med., 2013. 7(9): p. 729-741.
241. Su, R.J., et al., Efficient Generation of Integration-Free iPS Cells from Human Adult Peripheral Blood Using BCL-XL Together with Yamanaka Factors. PLoS ONE, 2013. 8(5).

242. Su, R.J., et al., Few single nucleotide variations in exomes of human cord blood induced pluripotent stem cells. PloS one, 2013. 8(4): p. e59908.
243. Teo, A.K., et al., Derivation of human induced pluripotent stem cells from patients with maturity onset diabetes of the young. The Journal of biological chemistry, 2013. 288(8): p. 5353-6.
244. Tiscornia, G., et al., Neuronopathic Gaucher's disease: Induced pluripotent stem cells for disease modelling and testing chaperone activity of small compounds. Hum. Mol. Genet., 2013. 22(4): p. 633-645.
245. Trilck, M., Niemann-Pick type C1 patient-specific induced pluripotent stem cells display disease specific hallmarks. Orphanet Journal of Rare diseases, 2013. 8(144)
246. Trokovic, R., et al., Small molecule inhibitors promote efficient generation of induced pluripotent stem cells from human skeletal myoblasts. Stem cells and development, 2013. 22(1): p. 114-23.
247. Tucker, B.A., et al., Patient-Specific Induced Pluripotent Stem Cells (IPSCS) Allow Direct Investigation of Disease Mechanisms in Inherited Retinal Degeneration. Trans. Am. Ophthalmol. Soc., 2013. 111: p. 20.
248. Wang, Y.M., et al., Induced Pluripotent Stem Cells from Human Hair Follicle Mesenchymal Stem Cells. Stem cell reviews and reports, 2013. 9(4): p. 451-460.
249. Wen, Y., et al., Reprogramming of fibroblasts from older women with pelvic floor disorders alters cellular behavior associated with donor age. Vol. 2. 2013. 118-28.
250. Yung, S.K., et al., Brief report: Human pluripotent stem cell models of fanconi anemia deficiency reveal an important role for fanconi anemia proteins in cellular reprogramming and survival of hematopoietic progenitors. Stem Cells, 2013. 31(5): p. 1022-1029.
251. Zhang, L., et al., Human bone marrow mesenchymal stem cells support the derivation and propagation of human induced pluripotent stem cells in culture. Vol. 15. 2013. 216-23.
252. Zhou, J., et al., Generation and characterization of human cryptorchid-specific induced pluripotent stem cells from urine. Stem cells and development, 2013. 22(5): p. 717-25.
253. Abdouh, M., et al., Transfer of malignant trait to immortalized human cells following exposure to human cancer serum. Journal of experimental & clinical cancer research : CR, 2014. 33(1): p. 86.
254. Basma, H., et al., Reprogramming of COPD lung fibroblasts through formation of induced pluripotent stem cells. American journal of physiology. Lung cellular and molecular physiology, 2014. 306(6): p. L552-65.
255. Ben-David, U., et al., Aneuploidy induces profound changes in gene expression, proliferation and tumorigenicity of human pluripotent stem cells. Nature communications, 2014. 5: p. 4825.
256. Chen, A.K., et al., Inhibition of ROCK-myosin II signaling pathway enables culturing of human pluripotent stem cells on microcarriers without extracellular matrix coating. Tissue engineering. Part C, Methods, 2014. 20(3): p. 227-38.
257. Durruthy-Durruthy, J., et al., Rapid and efficient conversion of integration-free human induced pluripotent stem cells to GMP-grade culture conditions. PloS one, 2014. 9(4): p. e94231.
258. Fu, X., et al., Genetic approach to track neural cell fate decisions using human embryonic stem cells. Protein & cell, 2014. 5(1): p. 69-79.
259. Fukawatase, Y., et al., Ataxia telangiectasia derived iPS cells show preserved x-ray sensitivity and decreased chromosomal instability. Scientific reports, 2014. 4: p. 5421.
260. Guan, X., et al., Dystrophin-deficient cardiomyocytes derived from human urine: new biologic reagents for drug discovery. Stem cell research, 2014. 12(2): p. 467-80.
261. Hansel, M.C., et al., Increased reprogramming of human fetal hepatocytes compared with adult hepatocytes in feeder-free conditions. Cell transplantation, 2014. 23(1): p. 27-38.
262. Hemmi, N., et al., A Massive Suspension Culture System With Metabolic Purification for Human Pluripotent Stem Cell-Derived Cardiomyocytes. Stem cells translational medicine, 2014. 3(12): p. 1473-1483.
263. Hibaoui, Y., et al., Modelling and rescuing neurodevelopmental defect of Down syndrome using induced pluripotent stem cells from mo0zygotic twins discordant for trisomy 21. EMBO molecular medicine, 2014. 6(2): p. 259-77.
264. Hosoi, M., et al., Generation of induced pluripotent stem cells derived from primary and secondary myelofibrosis patient samples. Vol. 42. 2014, Netherlands. 816-25.
265. Ichida, J.K., et al., notch inhibition allows oncogene-independent generation of iPS cells. Nat. Chem. Biol., 2014. 10(8): p. 632-639.
266. Kobayashi, J., et al. Directed differentiation of patient-specific induced pluripotent stem cells identifies the cardiac transcriptional repression during myocardial growth and patterning in hypoplastic left heart syndrome. PLoS One, 2014.
267. Lee, J., et al., Generation of disease-specific induced pluripotent stem cells from patients with rheumatoid arthritis and osteoarthritis. Arthritis research & therapy, 2014. 16(1): p. R41.

268. Liu, J., et al., Integration-Free Human Induced Pluripotent Stem Cells From Type 1 Diabetes Patient Skin Fibroblasts Show Increased Abundance of Pancreas-Specific microRNAs. Cell medicine, 2014. 7(1): p. 15-24.
269. Luo, Y.Q., et al., Stable Enhanced Green Fluorescent Protein Expression After Differentiation and Transplantation of Reporter Human Induced Pluripotent Stem Cells Generated by AAVS1 Transcription Activator-Like Effector Nucleases. Stem cells translational medicine, 2014. 3(7): p. 821-835.
270. Mitchell, R.R., et al., Activation of neural cell fate programs toward direct conversion of adult human fibroblasts into tri-potent neural progenitors using OCT-4. Stem cells and development, 2014. 23(16): p. 1937-46.
271. Numasawa-Kuroiwa, Y., et al., Involvement of ER stress in dysmyelination of Pelizaeus-Merzbacher Disease with PLP1 missense mutations shown by iPSC-derived oligodendrocytes. Stem cell reports, 2014. 2(5): p. 648-61.
272. Pipino, C., et al., Trisomy 21 mid-trimester amniotic fluid induced pluripotent stem cells maintain genetic signatures during reprogramming: implications for disease modeling and cryobanking. Cellular reprogramming, 2014. 16(5): p. 331-44.
273. Quang, T., et al., Dosage and cell line dependent inhibitory effect of bFGF supplement in human pluripotent stem cell culture on inactivated human mesenchymal stem cells. PloS one, 2014. 9(1): p. e86031.
274. Richards, M., et al., A New Class of Pluripotent Stem Cell Cytotoxic Small Molecules. Plos one, 2014. 9(3).
275. Spitalieri, P., et al., Generation of disease-specific induced pluripotent stem cells from human fetal extra-embryonic tissues. Cytotherapy, 2014. 16: p. S9.
276. Suzuki, D.E., A.M. Nakahata, and O.K. Okamoto, Knockdown of E2F2 inhibits tumorigenicity, but preserves stemness of human embryonic stem cells. Stem cells and development, 2014. 23(11): p. 1266-74.
277. Suzuki, T., et al., Use of induced pluripotent stem cells to recapitulate pulmonary alveolar protei0sis pathogenesis. American journal of respiratory and critical care medicine, 2014. 189(2): p. 183-93.
278. Takashima, Y., et al., Resetting transcription factor control circuitry toward ground-state pluripotency in human. Cell, 2014. 158(6): p. 1254-1269.
279. Tan, H.K., et al., Human finger-prick induced pluripotent stem cells facilitate the development of stem cell banking. Stem Cells Transl. Med., 2014. 3(5): p. 586-598.
280. Varela, I., et al., Generation of human β-thalassemia induced pluripotent cell lines by reprogramming of bone marrow-derived mesenchymal stromal cells using modified mRNA. Cellular reprogramming, 2014. 16(6): p. 447-55.
281. Ware, C.B., et al., Derivation of naïve human embryonic stem cells. Proc. Natl. Acad. Sci. U. S. A., 2014. 111(12): p. 4484-4489.
282. Yamasaki, S., et al., Generation of human induced pluripotent stem (Ips) cells in serum- and feeder-free defined culture and TGF-B1 regulation of pluripotency. PloS one, 2014. 9(1): p. e87151.
283. Zhao, L., T. Teklemariam, and B.M. Hantash, Heterelogous expression of mutated HLA-G decreases immunogenicity of human embryonic stem cells and their epidermal derivatives. Stem cell research, 2014. 13(2): p. 342-54.
284. Zhu, F.L., et al., A Modified Method for Implantation of Pluripotent Stem Cells Under the Rodent Kidney Capsule. Stem cells and development, 2014. 23(17): p. 2119-2125.
285. Assou, S., et al., Cultured Cells from the Human Oocyte Cumulus Niche Are Efficient Feeders to Propagate Pluripotent Stem Cells. Stem Cells Dev., 2015. 24(19): p. 2317-2327.
286. Bazley, F.A., et al., Direct Reprogramming of Human Primordial Germ Cells into Induced Pluripotent Stem Cells: Efficient Generation of Genetically Engineered Germ Cells. Stem cells and development, 2015. 24(22): p. 2634-48.
287. Cabrera, S., et al., Generation of human iPSC line GRX-MCiPS4F-A2 from adult peripheral blood mononuclear cells (PBMCs) with Spanish genetic background. Stem cell research, 2015. 15(2): p. 337-40.
288. Cary, W.A., et al., Efficient Generation of Induced Pluripotent Stem and Neural Progenitor Cells From Acutely Harvested Dura Mater Obtained During Ventriculoperitoneal Shunt Surgery. 2015: United States. p. 1256-66.e1.
289. Chang, E.A., et al., Derivation of Ethnically Diverse Human Induced Pluripotent Stem Cell Lines. Scientific reports, 2015. 5.
290. Driscoll, C.B., et al., Nuclear reprogramming with a non-integrating human RNA virus. Stem Cell Res. Ther., 2015. 6(1).
291. Garitaonandia, I., et al., Increased risk of genetic and epigenetic instability in human embryonic stem cells associated with specific culture conditions. PloS one, 2015. 10(2): p. e0118307.
292. Ghasemi-Dehkordi, P., et al., Comparison between the cultures of human induced pluripotent stem cells (hhiPSc) on feeder-and serum-free system (Matrigel matrix), MEF and HDF feeder cell lines. Journal of cell communication and signaling, 2015. 9(3): p. 233-46.

293. Hu, Z., et al., Generation of Naivetropic Induced Pluripotent Stem Cells from Parkinson's Disease Patients for High-Efficiency Genetic Manipulation and Disease Modeling. Stem cells and development, 2015. 24(21): p. 2591-604.
294. Huang, B., et al., Establishment of human-embryonic-stem-cell line from mosaic trisomy 9 embryo. Taiwanese journal of obstetrics & gynecology, 2015. 54(5): p. 505-11.
295. Huang, B.X., et al., Etha0l Inactivated Mouse Embryonic Fibroblasts Maintain the Self-Renew and Proliferation of Human Embryonic Stem Cells. Plos one, 2015. 10(6).
296. Joddar, B., et al., Chemically fixed autologous feeder cell-derived niche for human induced pluripotent stem cell culture. Journal of materials chemistry. B, 2015. 3(11): p. 2301-2307.
297. Lai, D., et al., Derivation and characterization of human embryonic stem cells on human amnion epithelial cells. Scientific reports, 2015. 5: p. 10014.
298. Li, J., et al., Thymic derived iPs cells can be differentiated into cardiomyocytes. Frontiers in bioscience (Landmark edition), 2015. 20: p. 964-74.
299. Mitsui, K., et al., Conditionally replicating ade0virus prevents pluripotent stem cell-derived teratoma by specifically eliminating undifferentiated cells. Molecular therapy. Methods & clinical development, 2015. 2: p. 15026.
300. Okada, M., et al., Modeling type II collagenopathy skeletal dysplasia by directed conversion and induced pluripotent stem cells. Human molecular genetics, 2015. 24(2): p. 299-313.
301. Salci, K.R., et al., Derivation of human induced pluripotent stem cells through continued exposure of OCT4-induced plastic human fibroblasts to reprogramming media. Stem cell research, 2015. 15(1): p. 240-2.
302. Salci, K.R., et al., Acquisition of pluripotency through continued environmental influence on OCT4-induced plastic human fibroblasts. Stem cell research, 2015. 15(1): p. 221-30.
303. Steichen, C., et al., An Atypical Human Induced Pluripotent Stem Cell Line With a Complex, Stable, and Balanced Genomic Rearrangement Including a Large De 0vo 1q Uniparental Disomy. Stem cells translational medicine, 2015. 4(3): p. 224-229.
304. Tosca, L., et al., Genomic instability of human embryonic stem cell lines using different passaging culture methods. Molecular cytogenetics, 2015. 8: p. 30.
305. Tsankov, A.M., et al., A qPCRCR ScoreCard quantifies the differentiation potential of human pluripotent stem cells. Nature biotechnology, 2015. 33(11): p. 1182-U117.
306. Umezaki, Y., et al., Human Gingival Integration-Free hiPSc; a Source for MSC-Like Cells. International journal of molecular sciences, 2015. 16(6): p. 13633-48.
307. Wang, D., et al., Targeted Disruption of the β2-Microglobulin Gene Minimizes the Immunogenicity of Human Embryonic Stem Cells. Stem cells translational medicine, 2015. 4(10): p. 1234-45.
308. Wang, J., et al., Generation of clinical-grade human induced pluripotent stem cells in Xeno-free conditions. Stem Cell Res. Ther., 2015. 6(1).
309. Wang, X.M., et al., Induced pluripotent stem cell models of Zellweger spectrum disorder show impaired peroxisome assembly and cell type-specific lipid abnormalities. Stem cell research & therapy, 2015. 6: p. 158.
310. Yagyu, S., et al., An Inducible Caspase-9 Suicide Gene to Improve the Safety of Therapy Using Human Induced Pluripotent Stem Cells. Molecular therapy : the journal of the American Society of Gene Therapy, 2015. 23(9): p. 1475-85.
311. Yang, Y., et al., Heightened potency of human pluripotent stem cell lines created by transient BMP4 exposure. Proceedings of the National Academy of Sciences of the United States of America, 2015. 112(18): p. E2337-46.
312. Zhang, S.Z., et al., Urine-derived induced pluripotent stem cells as a modeling tool for paroxysmal kinesigenic dyskinesia. Biology open, 2015. 4(12): p. 1744-52.
313. Akutsu, H., et al., In vivo maturation of human embryonic stem cell-derived teratoma over time. Regen. Ther., 2016. 5: p. 31-39.
314. Amir, H., et al., Spontaneous single-copy loss of TP53 in human embryonic stem cells markedly increases cell proliferation and survival. Fertil. Steril., 2016. 106: p. e363-e364.
315. Bedel, A., et al., Preventing teratoma risks to the application of induced pluripotent stem cells use in hematology. Human gene therapy, 2016. 27(11): p. A96-A97.
316. Bhatt, N., et al., Robust reprogramming of Ataxia-Telangiectasia patient and carrier erythroid cells to induced pluripotent stem cells. Stem cell research, 2016. 17(2): p. 296-305.
317. Burnett, L.C., et al., Induced pluripotent stem cells (iPSC) created from skin fibroblasts of patients with Prader-Willi syndrome (PWS) retain the molecular signature of PWS. Stem cell research, 2016. 17(3): p. 526-530.
318. Chang, W.F., et al., Derivation of Patient Specific Pluripotent Stem Cells Using Clinically Discarded Cumulus Cells. PloS one, 2016. 11(11): p. e0165715.

319. Chao, M.P., et al., Pluripotent reprogramming of human AML resets leukemic behavior and models therapeutic targeting of subclones. Blood, 2016. 128(22).
320. Choi, S.W., et al., STB-HO, a novel mica fine particle, inhibits the teratoma-forming ability of human embryonic stem cells after in vivo transplantation. Oncotarget, 2016. 7(3): p. 2684-95.
321. De Sousa, P.A., et al., Derivation of the human embryonic stem cell line RCM1. Stem cell research, 2016. 16(2): p. 476-80.
322. Gao, Y., et al., Genome therapy of myotonic dystrophy type 1 iPSC cells for development of autologous stem cell therapy. Molecular Therapy, 2016. 24(8):1378-87.
323. Gerami-Naini, B., et al., Generation of Induced Pluripotent Stem Cells from Diabetic Foot Ulcer Fibroblasts Using a nonintegrative Sendai Virus. Cellular reprogramming, 2016. 18(4): p. 214-23.
324. Hadoux, J., et al., Generation of patient-specific and genome-edited MEN2A patient-derived induced pluripotent stem cells as novel tools for drug screening. Cancer Res., 2016. 76(14).
325. Huynh, L.M., T. Shinagawa, and S. Ishii, Two Histone Variants TH2A and TH2B Enhance Human Induced Pluripotent Stem Cell Generation. Stem cells and development, 2016. 25(3): p. 251-8.
326. Jin, H., et al., Increased activity of TNAP compensates for reduced ade0sine production and promotes ectopic calcification in the genetic disease ACDC. Science signaling, 2016. 9(458): p. ra121.
327. Kamada, M., et al., Reversible transformation and de-differentiation of human cells derived from induced pluripotent stem cell teratomas. Human cell, 2016. 29(1): p. 1-9.
328. Kao, T., et al., GAPTrap: A Simple Expression System for Pluripotent Stem Cells and Their Derivatives. Stem cell reports, 2016. 7(3): p. 518-526.
329. Kotini, A.G., et al., Escape Mutations, Ganciclovir Resistance, and Teratoma Formation in Human iPSCs Expressing an HSVtk Suicide Gene. Mol. Ther. Nucl. Acids, 2016. 5: p. e284.
330. Kotini, A.G., M. Sadelain, and E.P. Papapetrou, LiPS-A3S, a human genomic site for robust expression of inserted transgenes. Mol. Ther. Nucl. Acids, 2016. 5: p. e394.
331. Kyrkou, A., et al., Generation of human induced pluripotent stem cells in defined, feeder-free conditions. Stem cell research, 2016. 17(2): p. 458-460.
332. Li, Y., et al., Reprogramming of blood cells into induced pluripotent stem cells as a new cell source for cartilage repair. Stem cell research & therapy, 2016. 7: p. 31.
333. Liang, D., et al., Generation of MERRF patient-derived induced pluripotent stem cell line iMERRF-C7. Stem cell research, 2016. 17(3): p. 616-618.
334. Liu, C.C., et al., Distinct Responses of Stem Cells to Telomere Uncapping-A Potential Strategy to Improve the Safety of Cell Therapy. Stem cells (Dayton, Ohio), 2016. 34(10): p. 2471-2484.
335. Liu, L., et al., Generation of human embryonic stem cell line chHES-472 from abnormal embryos diagnosed with Spi0cerebellar ataxia type 3. Stem cell research, 2016. 17(3): p. 584-586.
336. Lopez-Onieva, L., et al., Generation of a human induced pluripotent stem cell (iPSC) line from a Bernard-Soulier syndrome patient with the mutation p.Asn45Ser in the GPIX gene. Stem cell research, 2016. 17(3): p. 603-606.
337. Maguire, J.A., et al., Generation of Hermansky-Pudlak Syndrome Type 1 (HPS1) induced pluripotent stem cells (iPSCs). Stem cell research, 2016. 16(2): p. 233-5.
338. Mori, E., et al., Impaired adipogenic capacity in induced pluripotent stem cells from lipodystrophic patients with BSCL2 mutations. Metabolism: clinical and experimental, 2016. 65(4): p. 543-56.
339. Ouyang, Q., et al., Human embryonic stem cells derived from abnormal blastocyst donated by polycystic kidney syndrome patient. Stem cell research, 2016. 17(3): p. 637-639.
340. Parr, C.J., et al., MicroRNA-302 switch to identify and eliminate undifferentiated human pluripotent stem cells. Scientific reports, 2016. 6: p. 32532.
341. Phondeechareon, T., et al., Generation of induced pluripotent stem cells as a potential source of hematopoietic stem cells for transplant in PNH patients. Vol. 95. 2016, Germany. 1617-25.
342. Qin, H., et al., YAP Induces Human Naive Pluripotency. Cell Rep., 2016. 14(10): p. 2301-2312.
343. Qin, M., et al., Direct Reprogramming of Human Amniotic Fluid Stem Cells by OCT4 and Application in Repairing of Cerebral Ischemia Damage. International journal of biological sciences, 2016. 12(5): p. 558-68.
344. Rohani, L., et al., Generation of human induced pluripotent stem cells using non-synthetic mRNA. Stem cell research, 2016. 16(3): p. 662-72.
345. Shimizu, T., et al., Derivation of integration-free iPSCs from a Klinefelter syndrome patient. Reproductive medicine and biology, 2016. 15(1): p. 35-43.
346. Slamecka, J., et al., non-integrating episomal plasmid-based reprogramming of human amniotic fluid stem cells into induced pluripotent stem cells in chemically defined conditions. Cell cycle (Georgetown, Tex.), 2016. 15(2): p. 234-49.
347. Sun, Y., et al., Generation of human embryonic stem cells from abnormal blastocyst diagnosed with albinism. Stem cell research, 2016. 17(3): p. 643-645.

348. Telliam, G., et al., Generation of an induced pluripotent stem cell line from a patient with chronic myeloid leukemia (CML) resistant to targeted therapies. Stem cell research, 2016. 17(2): p. 235-237.
349. Unzu, C., et al., Human Hepatocyte-Derived Induced Pluripotent Stem Cells: MYC Expression, Similarities to Human Germ Cell Tumors, and Safety Issues. Stem cells international, 2016. 2016: p. 4370142.
350. Wang, J., et al., Differentiation of human foreskin fibroblast-derived induced pluripotent stem cells into hepatocyte-like cells. Cell biochemistry and function, 2016. 34(7): p. 475-482.
351. Yamasaki, S., et al., Generation of cleidocranial dysplasia-specific human induced pluripotent stem cells in completely serum-, feeder-, and integration-free culture. In vitro cellular & developmental biology. Animal, 2016. 52(2): p. 252-64.
352. Yan, H.J., et al., The effects of LSD1 inhibition on self-renewal and differentiation of human induced pluripotent stem cells. Exp. Cell Res., 2016. 340(2): p. 227-237.
353. Yao, L., et al., Generation of induced pluripotent stem cells with high efficiency from human embryonic renal cortical cells. American journal of translational research, 2016. 8(11): p. 4982-4993.
354. Yin, X., et al., Generation and periodontal differentiation of human gingival fibroblasts-derived integration-free induced pluripotent stem cells. Biochemical and biophysical research communications, 2016. 473(3): p. 726-32.
355. Zhang, D., et al., Comparison of a xeno-free and serum-free culture system for human embryonic stem cells with conventional culture systems. Stem Cell Res. Ther., 2016. 7(1).
356. Zhang, Q., et al., Role of Jnk1 in development of neural precursors revealed by iPSC modeling. Oncotarget, 2016. 7(38): p. 60919-60928.
357. Avior, Y., et al., Modeling Developmental and Tumorigenic Aspects of Trilateral Reti0blastoma via Human Embryonic Stem Cells. Stem cell reports, 2017. 8(5): p. 1354-1365.
358. Barry, C., et al., Species-specific developmental timing is maintained by pluripotent stem cells ex utero. Developmental biology, 2017. 423(2): p. 101-110.
359. Bennaceur-Griscelli, A., et al., Generation of an induced pluripotent stem cell line from a patient with hereditary multiple endocrine neoplasia 2B (MEN2B) syndrome with "highest risk" RET mutation. Stem cell research, 2017. 23: p. 154-157.
360. Bouma, M.J., et al., Differentiation-Defective Human Induced Pluripotent Stem Cells Reveal Strengths and Limitations of the Teratoma Assay and In Vitro Pluripotency Assays. Stem cell reports, 2017. 8(5): p. 1340-1353.
361. Chang, R., et al., Na0porous Immunoprotective Device for Stem-Cell-Derived beta-Cell Replacement Therapy. Acs na0, 2017. 11(8): p. 7747-7757.
362. Choi, Y.J., et al., Recombination activating gene-2(null) severe combined immunodeficient pigs and mice engraft human induced pluripotent stem cells differently. Oncotarget, 2017. 8(41): p. 69398-69407.
363. Hasegawa, D., et al., Gorlin syndrome-derived induced pluripotent stem cells are hypersensitive to hedgehog-mediated osteogenic induction. PloS one, 2017. 12(10): p. e0186879.
364. Horikawa, I., et al., Δ133p53 represses p53-inducible senescence genes and enhances the generation of human induced pluripotent stem cells. Cell Death Differ., 2017. 24(6): p. 1017-1028.
365. Hu, K., et al., Generation of an induced pluripotent stem cell line from a Loeys-Dietz syndrome patient with transforming growth factor-beta receptor-2 gene mutation. Stem cell research, 2017. 20: p. 115-117.
366. Hu, L., et al., Modeling Glanzmann thrombasthenia using patient specific iPSCs and restoring platelet aggregation function by CD41 overexpression. Stem cell research, 2017. 20: p. 14-20.
367. Jiang, Z., et al., Protein profiling identified key chemokines that regulate the maintenance of human pluripotent stem cells. Scientific reports, 2017. 7(1): p. 14510.
368. Khudiakov, A., et al., Generation of iPSC line from desmin-related cardiomyopathy patient carrying splice site mutation of DES gene. Stem Cell Res., 2017. 24: p. 77-80.
369. Khudiakov, A., et al., Generation of iPSC line from patient with arrhythmogenic right ventricular cardiomyopathy carrying mutations in PKP2 gene. 2017: England. p. 85-88.
370. Kim, Y.M., et al., Effects of mechanical stimulation on the reprogramming of somatic cells into human-induced pluripotent stem cells. Stem cell research & therapy, 2017. 8(1): p. 139.
371. Kuang, Y., et al., Efficient, Selective Removal of Human Pluripotent Stem Cells via Ecto-Alkaline Phosphatase-Mediated Aggregation of Synthetic Peptides. Cell chemical biology, 2017. 24(6): p. 685-694.e4.
372. Lee, A.S., et al., Brief Report: External Beam Radiation Therapy for the Treatment of Human Pluripotent Stem Cell-Derived Teratomas. Stem cells (Dayton, Ohio), 2017. 35(8): p. 1994-2000.
373. Lee, J.H., et al., Lineage-Specific Differentiation Is Influenced by State of Human Pluripotency. Cell reports, 2017. 19(1): p. 20-35.
374. Lee, S.B., et al., Post-irradiation promotes susceptibility to reprogramming to pluripotent state in human fibroblasts. Cell cycle (Georgetown, Tex.), 2017. 16(21): p. 2119-2127.

375. Li, Y., et al., Targeted Disruption of TCF12 Reveals HEB as Essential in Human Mesodermal Specification and Hematopoiesis. Stem Cell Rep., 2017. 9(3): p. 779-795.
376. Lin, Y.T., et al., Elimination of undifferentiated human embryonic stem cells by cardiac glycosides. Scientific reports, 2017. 7(1): p. 5289.
377. Ma, D., et al., Development of a human induced pluripotent stem cell (iPSC) line from a Parkinson's disease patient carrying the N551K variant in LRRK2 gene. Stem cell research, 2017. 18: p. 51-53.
378. Ma, D., et al., Generation of a human induced pluripotent stem cell (iPSC) line carrying the Parkinson's disease linked LRRK2 variant S1647T. Stem cell research, 2017. 18: p. 54-56.
379. Ma, D., et al., Derivation of human induced pluripotent stem cell (iPSC) line with LRRK2 gene R1398H variant in Parkinson's disease. Stem cell research, 2017. 18: p. 48-50.
380. Miyagoe-Suzuki, Y., et al., Induction of Pluripotent Stem Cells from a Manifesting Carrier of Duchenne Muscular Dystrophy and Characterization of Their X-Inactivation Status. Stem cells international, 2017. 2017: p. 7906843.
381. Nayler, S., et al., Lentiviral Reprogramming of A-T Patient Fibroblasts to Induced Pluripotent Stem Cells. Methods in molecular biology (Clifton, N.J.), 2017. 1599: p. 401-418.
382. Ota, A., et al., StemCell Keep™ Is Effective for Cryopreservation of Human Embryonic Stem Cells by Vitrification. Cell transplantation, 2017. 26(5): p. 773-787.
383. Romero-Moya, D., et al., Generation, genome edition and characterization of iPSC lines from a patient with coenzyme Q(10) deficiency harboring a heterozygous mutation in COQ4 gene. 2017: England. p. 144-147.
384. Rosner, M., et al., Human stem cells alter the invasive properties of somatic cells via paracrine activation of mTORC1. Nature communications, 2017. 8(1): p. 595.
385. Schmitt, C.E., et al., Fluorescent tagged episomals for stoichiometric induced pluripotent stem cell reprogramming. Stem Cell Res. Ther., 2017. 8(1).
386. Shin, D.H., et al., Characterization of Tetraploid Somatic Cell Nuclear Transfer-Derived Human Embryonic Stem Cells. Development & reproduction, 2017. 21(4): p. 425-434.
387. Shukla, V., et al., ASXL3 Is a novel Pluripotency Factor in Human Respiratory Epithelial Cells and a Potential Therapeutic Target in Small Cell Lung Cancer. Cancer research, 2017. 77(22): p. 6267-6281.
388. Slamecka, J., et al., Reprogramming Primary Amniotic Fluid and Membrane Cells to Pluripotency in Xeno-free Conditions. Journal of visualized experiments : JoVE, 2017(129).
389. Velasquez-Mao, A.J., et al., Differentiation of spontaneously contracting cardiomyocytes from non-virally reprogrammed human amniotic fluid stem cells. PloS one, 2017. 12(5): p. e0177824.
390. Wang, C.K., et al., CHAC2 is essential for self-renewal and glutathione maintenance in human embryonic stem cells. Free radical biology & medicine, 2017. 113: p. 439-451.
391. Wolfs, E., et al., Molecular Imaging of Human Embryonic Stem Cells Stably Expressing Human PET Reporter Genes After Zinc Finger Nuclease-Mediated Genome Editing. Journal of nuclear medicine : official publication, Society of Nuclear Medicine, 2017. 58(10): p. 1659-1665.
392. Yang, M., et al., Mitomycin C-treated human-induced pluripotent stem cells as a safe delivery system of gold na0rods for targeted photothermal therapy of gastric cancer. Na0scale, 2017. 9(1): p. 334-340.
393. Zhao, Y., et al., Induction of reprogramming of human amniotic epithelial cells into iPS cells by overexpression of Yap, Oct4, and Sox2 through the activation of the Hippo-Yap pathway. Experimental and therapeutic medicine, 2017. 14(1): p. 199-206.
394. Zhu, L.F., et al., The Construction and Identification of Induced Pluripotent Stem Cells Derived from Acute Myelogenous Leukemia Cells. Cellular physiology and biochemistry : international journal of experimental cellular physiology, biochemistry, and pharmacology, 2017. 41(4): p. 1661-1674.
395. Zhu, Y., et al., The generation and functional characterization of induced pluripotent stem cells from human intervertebral disc nucleus pulposus cells. Oncotarget, 2017. 8(26) 42700-42711.
396. Zhu, Z., et al., Establishment of an induced pluripotent stem cell line ZZUi003-A from a 65-year-old male with sporadic Parkinson's disease. Stem Cell Res., 2017. 23: p. 119-121.
397. Bidollari, E., et al., Generation of induced pluripotent stem cell line, CSSi004-A (2962), from a patient diagnosed with Huntington's disease at the presymptomatic stage. Stem cell research, 2018. 28: p. 145-148.
398. Cao, Y., et al., Generation of a Urine-Derived Ips Cell Line from a Patient with a Ventricular Septal Defect and Heart Failure and the Robust Differentiation of These Cells to Cardiomyocytes via Small Molecules. Cellular physiology and biochemistry : international journal of experimental cellular physiology, biochemistry, and pharmacology, 2018. 50(2): p. 538-551.
399. Capuano, R., et al., Volatile compounds emission from teratogenic human pluripotent stem cells observed during their differentiation in vivo. Scientific reports, 2018. 8(1): p. 11056.
400. Dejosez, M. and T.P. Zwaka, Generation of hiPSTZ16 (ISMMSi003-A) cell line from normal human foreskin fibroblasts. Stem cell research, 2018. 26: p. 44-46.

401. Fang, F., et al., Derivation of human induced pluripotent stem cell line (HUSTi001-A) from a 40-year-old patient with idiopathic infertility. Stem cell research, 2018. 27: p. 136-139.
402. Fernandes, S., et al., Derivation of human iPSC line NCCSi002-A from umbilical cord blood (UCB) CD34+cells of donor from Indian ethnicity. Stem cell research, 2018. 26: p. 80-83.
403. Fu, S., et al., Generation of human-induced pluripotent stem cells from burn patient-derived skin fibroblasts using a non-integrative method. International journal of molecular medicine, 2018. 41(1): p. 87-94.
404. Ide, K., et al., A novel Construction of Lentiviral Vectors for Eliminating Tumorigenic Cells from Pluripotent Stem Cells. Stem cells (Dayton, Ohio), 2018. 36(2): p. 230-239.
405. Kahounová, Z., et al., Generation of human iPSCs from human prostate cancer-associated fibroblasts IBPi002-A. 2018: England. p. 255-259.
406. Lambshead, J.W., et al., Long-Term Maintenance of Human Pluripotent Stem Cells on cRGDfK-Presenting Synthetic Surfaces. Scientific reports, 2018. 8(1): p. 701.
407. Matsumura, W., et al., Establishment of integration-free induced pluripotent stem cells from human recessive dystrophic epidermolysis bullosa keratinocytes. Journal of dermatological science, 2018. 89(3): p. 263-271.
408. Min, Z.Y., et al., New insights into the genic and metabolic characteristics of induced pluripotent stem cells from polycystic ovary syndrome women. Stem cell research & therapy, 2018. 9.
409. Nakashima, Y., et al., Atorvastatin Inhibits the HIF1$\alpha$-PPAR Axis, Which Is Essential for Maintaining the Function of Human Induced Pluripotent Stem Cells. Molecular therapy : the journal of the American Society of Gene Therapy, 2018. 26(7): p. 1715-1734.
410. Ohmine, S., et al., Aurora-A overexpression is linked to development of aggressive teratomas derived from human iPS cells. Oncology reports, 2018. 39(4): p. 1725-1730.
411. Osada, N., et al., Lysine-specific demethylase 1 inhibitors prevent teratoma development from human induced pluripotent stem cells. Oncotarget, 2018. 9(5): p. 6450-6462.
412. Pan, L., et al., Quantitative Proteomics Study Reveals Changes in the Molecular Landscape of Human Embryonic Stem Cells with Impaired Stem Cell Differentiation upon Exposure to Titanium Dioxide Na0particles. Small (Weinheim an der Bergstrasse, Germany), 2018. 14(23): p. e1800190.
413. Park, J., et al., DSG2 Is a Functional Cell Surface Marker for Identification and Isolation of Human Pluripotent Stem Cells. Stem cell reports, 2018. 11(1): p. 115-127.
414. Parveen, S., Establishment and characterization of induced pluripotent stem cells from placental mesenchymal stromal cells. Stem Cell Res., 2018. 27: p. 15-20.
415. Russell, O.M., et al., Preferential amplification of a human mitochondrial DNA deletion in vitro and in vivo. Sci Rep, 2018. 8(1): p. 1799.
416. Salvatori, D.C.F., et al., The MicroRNA-371 Family as Plasma Biomarkers for Monitoring Undifferentiated and Potentially Malignant Human Pluripotent Stem Cells in Teratoma Assays. Stem cell reports, 2018. 11(6): p. 1493-1505.
417. Schick, R., et al., Functional abnormalities in induced pluripotent stem cell-derived cardiomyocytes generated from titin-mutated patients with dilated cardiomyopathy. PLOS one, 2018. 13(10).
418. Slamecka, J., et al., Induced pluripotent stem cells derived from human amnion in chemically defined conditions. Cell cycle (Georgetown, Tex.), 2018. 17(3): p. 330-347.
419. Taoka, K., et al., Using patient-derived iPSCs to develop humanized mouse models for chronic myelomo0cytic leukemia and therapeutic drug identification, including liposomal clodronate. Scientific reports, 2018. 8(1): p. 15855.
420. Wang, H., et al., Generation of integration-free induced pluripotent stem cells from a patient with spina bifida. 2018: England. p. 27-30.
421. Wang, Y., et al., Establishment of an induced pluripotent stem cell model of Hirschsrpung disease, a congenital condition of the enteric nervous system, from a patient carrying a novel RET mutation. Neuroreport, 2018. 29(12): p. 975-980.
422. Yang, Y.H., et al., Generation of Induced Pluripotent Stem Cells from Human Epidermal Keratinocytes. Cellular reprogramming, 2018. 20(6): p. 356-364.
423. Yasuda, S., et al., Tumorigenicity-associated characteristics of human iPS cell lines. PloS one, 2018. 13(10): p. e0205022.
424. Ye, H. and Q. Wang, Efficient Generation of non-Integration and Feeder-Free Induced Pluripotent Stem Cells from Human Peripheral Blood Cells by Sendai Virus. Cellular physiology and biochemistry : international journal of experimental cellular physiology, biochemistry, and pharmacology, 2018. 50(4): p. 1318-1331.
425. Zhang, R.R., et al., Human iPSC-Derived Posterior Gut Progenitors Are Expandable and Capable of Forming Gut and Liver Organoids. Stem cell reports, 2018. 10(3): p. 780-793.

426. Zhou, R., et al., A homozygous p53 R282W mutant human embryonic stem cell line generated using TALEN-mediated precise gene editing. Stem Cell Res., 2018. 27: p. 131-135.
427. Zhou, S., et al., Application of amphiphilic fluorophore-derived na0particles to provide contrast to human embryonic stem cells without affecting their pluripotency and to monitor their differentiation into neuron-like cells. Acta biomaterialia, 2018. 78: p. 274-284.
428. Altieri, F., et al., Production and characterization of human induced pluripotent stem cells (iPSC) CSSi007-A (4383) from Joubert Syndrome. Stem Cell Res., 2019. 38.
429. Arasala, R.R., et al., Characterization of new variant human ES line VH9 hESC (INSTEMe001-a): a tool for human stem cell and cancer research. Stem cell research, 2019. 37: p. 101444.
430. Ávila-González, D., et al., Establishment of human embryonic stem cell line Amicqui-2 using poor-quality embryos from Mexican population. Stem cell research, 2019. 34: p. 101364.
431. Baskfield, A., et al., Generation of an induced pluripotent stem cell line (TRNDi004-I) from a Niemann-Pick disease type B patient carrying a heterozygous mutation of p.L43_A44delLA in the SMPD1 gene. Stem cell research, 2019. 37: p. 101436.
432. Benabdallah, B., et al., Natural Killer Cells Prevent the Formation of Teratomas Derived From Human Induced Pluripotent Stem Cells. Frontiers in immunology, 2019. 10: p. 2580.
433. Carpenedo, R.L., et al., Transcriptomically Guided Mesendoderm Induction of Human Pluripotent Stem Cells Using a Systematically Defined Culture Scheme. Stem cell reports, 2019. 13(6): p. 1111-1125.
434. Cheng, Y.C., et al., Reprogramming of a human induced pluripotent stem cell (iPSC) line (IBMSi012-A) from an early-onset Parkinson's disease patient harboring a homozygous p.D331Y mutation in the PLA2G6 gene. Stem cell research, 2019. 37: p. 101432.
435. Cheng, Y.S., et al., A human induced pluripotent stem cell line (TRNDi007-B) from an infantile onset Pompe patient carrying p.R854X mutation in the GAA gene. Stem cell research, 2019. 37: p. 101435.
436. Han, H.J., et al., Generation of a TLR7 homozygous knockout human induced pluripotent stem cell line using CRISPR/Cas9. Stem cell research, 2019. 40: p. 101520.
437. Hong, J., et al., Generation of an induced pluripotent stem cell line (TRNDi008-A) from a Hunter syndrome patient carrying a hemizygous 208insC mutation in the IDS gene. Stem cell research, 2019. 37: p. 101451.
438. Huang, W., et al., An induced pluripotent stem cell line (TRNDi006-A) from a MPS IIIB patient carrying homozygous mutation of p.Glu153Lys in the NAGLU gene. Stem cell research, 2019. 37: p. 101427.
439. Huang, W.S., et al., Melanocyte Differentiation From Induced Pluripotent Stem Cells Derived From Human Adipose-Derived Stem Cells. Annals of plastic surgery, 2019. 82(1): p. S119-S125.
440. Huo, Z., et al., Generation of a heterozygous p53 R249S mutant human embryonic stem cell line by TALEN-mediated genome editing. Stem cell research, 2019. 34: p. 101360.
441. Kahounová, Z., et al., Generation of human iPSCs from fetal prostate fibroblasts HPrF. Stem cell research, 2019. 35: p. 101405.
442. Kerr, C.L., et al., Targeting RNA helicase DDX3 in stem cell maintenance and teratoma formation. Genes & cancer, 2019. 10(1): p. 11-20.
443. Laowtammathron, C., et al., Derivation of a MUSIi012-A iPSCs from mobilized peripheral blood stem cells. Stem cell research, 2019. 41: p. 101597.
444. Li, G., et al., Generation and Characterization of Induced Pluripotent Stem Cells and Retinal Organoids From a Leber's Congenital Amaurosis Patient With novel RPE65 Mutations. Frontiers in molecular neuroscience, 2019. 12: p. 212.
445. Lin, H.I., et al., Generation of induced pluripotent stem cells (IBMSi011-A) from a patient with Parkinson's disease carrying LRRK2 p.I1371V mutation. Stem cell research, 2019. 37: p. 101447.
446. Mao, S.H., C.H. Chen, and C.T. Chen, Osteogenic potential of induced pluripotent stem cells from human adipose-derived stem cells. Stem cell research & therapy, 2019. 10(1): p. 303.
447. Mitchell, A., et al., Assessment of iPSC teratogenicity throughout directed differentiation toward an alveolar-like phenotype. Differentiation; research in biological diversity, 2019. 105: p. 45-53.
448. Montes, R., et al., GENYOi004-A: An induced pluripotent stem cells (hiPSc) line generated from a patient with autism-related ADNP syndrome carrying a pTyr719* mutation. Stem cell research, 2019. 37: p. 101446.
449. Pandolfi, E.C., et al., Generation of three human induced pluripotent stem cell sublines (MZT04D, MZT04J, MZT04C) for reproductive science research. Stem cell research, 2019. 40: p. 101576.
450. Panula, S., et al., Human induced pluripotent stem cells from two azoospermic patients with Klinefelter syndrome show similar X chromosome inactivation behavior to female pluripotent stem cells. Human reproduction (Oxford, England), 2019. 34(11): p. 2297-2310.
451. Patterson, K., et al., Generation of two induced pluripotent stem cell lines (NHLBIi001-A and NHLBIi001-B) from a healthy Caucasian female volunteer with normal cardiac function. Stem Cell Res., 2019. 41.

452. Ravanpay, A.C., et al., EGFR806-CAR T cells selectively target a tumor-restricted EGFR epitope in glioblastoma. Oncotarget, 2019. 10(66): p. 7080-7095.
453. Tang, C., et al., Generation of two induced pluripotent stem cell (iPSC) lines from human breast milk using episomal reprogramming system. Stem cell research, 2019. 39: p. 101511.
454. Tangprasittipap, A., et al., Generation of a human induced pluripotent stem cell line (MUi010-A) from skin fibroblast of patient carrying a c.2104C>T mutation in MYH9 gene. Stem Cell Res., 2019. 36.
455. Wang, Y., et al., Generation of induced pluripotent stem cell line (ZZUi0014-A) from a patient with spi0cerebellar ataxia type 3. Stem Cell Res., 2019. 41.
456. 714. Weissbein, U., et al., Genome-wide Screen for Culture Adaptation and Tumorigenicity-Related Genes in Human Pluripotent Stem Cells. iScience, 2019. 11: p. 398-408.
457. Wilkinson, A.C., et al., Expanded potential stem cell media as a tool to study human developmental hematopoiesis in vitro. Vol. 76. 2019. 1-12.e5.
458. Wu, Y., et al., Using Gene Editing to Establish a Safeguard System for Pluripotent Stem-Cell-Based Therapies. iScience, 2019. 22: p. 409-422.
459. Yang, S., et al., An induced pluripotent stem cell line (TRNDi010-C) from a patient carrying a homozygous p.R401X mutation in the NGLY1 gene. Stem Cell Res., 2019. 39.
460. Cai, Y., et al., Generation of a human induced pluripotent stem cell line (SHUPLi001-A) from PBMCs of a healthy male donor. Stem Cell Res., 2020. 47.
461. Chlebanowska, P., et al., Origin of the Induced Pluripotent Stem Cells Affects Their Differentiation into Dopaminergic Neurons. International journal of molecular sciences, 2020. 21(16).
462. Choi, N.Y., et al., Generation of human androgenetic induced pluripotent stem cells. Scientific reports, 2020. 10(1): p. 3614.
463. Cui, P., et al., HIF-1$\alpha$/Actl6a/H3K9ac axis is critical for pluripotency and lineage differentiation of human induced pluripotent stem cells. FASEB journal : official publication of the Federation of American Societies for Experimental Biology, 2020. 34(4): p. 5740-5753.
464. Deng, Y., et al., METTL1-mediated m(7)G methylation maintains pluripotency in human stem cells and limits mesoderm differentiation and vascular development. Stem cell research & therapy, 2020. 11(1): p. 306.
465. Gao, Y., et al., Generation of induced pluripotent stem cell line (ZZUi0016-A) from dermal fibroblasts of a normal human. Stem Cell Res., 2020. 43.
466. Haramoto, Y., et al., A technique for removing tumorigenic pluripotent stem cells using rBC2LCN lectin. Regenerative therapy, 2020. 14: p. 306-314.
467. He, S., et al., Establishment of an induced pluripotent stem cell line from a patient with CHARGE syndrome carrying a CHD7 (p.L1151Gfs*17) mutation. Stem Cell Res., 2020. 45.
468. He, S., et al., Establishment of an induced pluripotent stem cell line PUMCHi004-A from a hereditary transthyretin amyloid cardiomyopathy patient with transthyretin (TTR) p.Asp38Asn mutation. Stem cell research, 2020. 49: p. 102022.
469. Huang, C.Y., et al., Establishment of an induced pluripotent stem cell (iPSC) line from a 7-year-old male patient with profound hearing loss carrying c.235delC in GJB2 gene. Stem Cell Res., 2020. 45.
470. Ikemoto, Y., et al., Gorlin syndrome-induced pluripotent stem cells form medulloblastoma with loss of heterozygosity in PTCH1. Aging, 2020. 12(10): p. 9935-9947.
471. Jamwal, V.S., et al., Generation of iPSC from fetal fibroblast cells obtained from an abortus with type-I tri-allelic variants. Stem cell research, 2020. 48: p. 101963.
472. Jo, H.Y., et al., Functional in vivo and in vitro effects of 20q11.21 genetic aberrations on hPSC differentiation. Scientific reports, 2020. 10(1): p. 18582.
473. Kuebler, B., et al., Derivation of induced pluripotent stem cells (hiPSc) by retroviral transduction of skin fibroblasts from four patients suffering 7q11.23 microduplication syndrome. Stem cell research, 2020. 49: p. 102092.
474. Lezmi, E., et al., The Chromatin Regulator ZMYM2 Restricts Human Pluripotent Stem Cell Growth and Is Essential for Teratoma Formation. Stem cell reports, 2020.
475. Li, H., et al., TET1 Deficiency Impairs Morphogen-free Differentiation of Human Embryonic Stem Cells to Neuroectoderm. Scientific reports, 2020. 10(1): p. 10343.
476. Long, P., et al., Generation of an induced pluripotent stem cell line from chorionic villi of a Patau syndrome spontaneous abortion. Stem cell research, 2020. 45: p. 101789.
477. Mui, B.W.H., et al., Generation of human induced pluripotent stem cell line (NIDCRi001-A) from a Muenke syndrome patient with an FGFR3 p.Pro250Arg mutation. Stem cell research, 2020. 46: p. 101823.
478. Park, T.S., et al., Vascular progenitors generated from tankyrase inhibitor-regulated naïve diabetic human iPSC potentiate efficient revascularization of ischemic retina. Nature communications, 2020. 11(1): p. 1195.

479. Shan, H., et al., Establishment of an induced pluripotent stem cell line from a patient with hereditary transthyretin amyloidosis carrying transthyretin (TTR) mutation p.Phe53Val. Stem cell research, 2020. 48: p. 101940.
480. Shi, Z.D., et al., Precision installation of a highly efficient suicide gene safety switch in human induced pluripotent stem cells. Stem cells translational medicine, 2020.
481. Sung, T.C., et al., Effect of cell culture biomaterials for completely xeno-free generation of human induced pluripotent stem cells. Biomaterials, 2020. 230.
482. Wang, G., et al., Generation of induced pluripotent stem cell line (NCKDi001-A) from a 19-year-old patient with a novel COL4A5 gene mutation in Alport syndrome. Stem cell research, 2020. 49: p. 102023.
483. Wang, H., et al., Role of CD133 in human embryonic stem cell proliferation and teratoma formation. Stem cell research & therapy, 2020. 11(1): p. 208.
484. Wang, Z., et al., Ultrasensitive and rapid quantification of rare tumorigenic stem cells in hPSC-derived cardiomyocyte populations. Science advances, 2020. 6(12): p. eaay7629.
485. Watanabe, K., et al., A novel GNAS-mutated human induced pluripotent stem cell model for understanding GNAS-mutated tumors. Tumor biology : the journal of the International Society for Oncodevelopmental Biology and Medicine, 2020. 42(9): p. 1010428320962588.
486. Xia, S., et al., Establishment of induced pluripotent stem cell lines from a family of an ARVC patient receiving heart transplantation in infant age carrying compound heterozygous mutations in DSP gene. Stem cell research, 2020. 48: p. 101977.
487. Yoshida, S., et al., Generation of four induced pluripotent stem cell lines (FHUi003-A, FHUi003-B, FHUi004-A and FHUi004-B) from two affected individuals of a familial neurohypophyseal diabetes insipidus family. Stem cell research, 2020. 48: p. 101960.
488. Zhang, Y., et al., Reprogramming Prostate Cancer Cells into Induced Pluripotent Stem Cells: a Promising Model of Prostate Cancer Stem Cell Research. Cellular reprogramming, 2020. 22(5): p. 262-268.
489. Zhao, H., et al., Generation of a tdTomato-GAD67 reporter human epilepsia mutation induced pluripotent stem cell line, USTCi001-A-2, using CRISPR/Cas9 editing. Stem cell research, 2020. 48: p. 102003.
490. Zhao, H., et al., Generation of iPSC line (USTCi001-A) from human skin fibroblasts of a patient with epilepsy. Stem Cell Res., 2020. 45.
491. Zhou, X.B., et al., Generation and characterization of two iPSC lines from human adipose tissue-derived stem cells of healthy donors. Stem cell research, 2020. 48: p. 101973.
492. Li, W., et al., Generation of systemic lupus erythematosus patient-derived induced pluripotent stem cells from blood. Stem cells and development, 2021.
